# Complex Fluid Models of Mixed Quantum–Classical Dynamics

**DOI:** 10.1007/s00332-024-10044-4

**Published:** 2024-07-02

**Authors:** François Gay-Balmaz, Cesare Tronci

**Affiliations:** 1 Division of Mathematical Sciences, Nanyang Technological University, Singapore, Singapore; 2 Department of Mathematics, University of Surrey, Guildford, UK

**Keywords:** Complex fluids, Mixed quantum-classical dynamics, Quantum backreaction, Euler-Poincaré reduction, Hamiltonian structure, 76A99, 82D15, 37N20, 81V55, 82M37, 53D20, 70H15, 37K58, 70H33

## Abstract

Several methods in nonadiabatic molecular dynamics are based on Madelung’s hydrodynamic description of nuclear motion, while the electronic component is treated as a finite-dimensional quantum system. In this context, the quantum potential leads to severe computational challenges and one often seeks to neglect its contribution, thereby approximating nuclear motion as classical. The resulting model couples classical hydrodynamics for the nuclei to the quantum motion of the electronic component, leading to the structure of a complex fluid system. This type of mixed quantum–classical fluid models has also appeared in solvation dynamics to describe the coupling between liquid solvents and the quantum solute molecule. While these approaches represent a promising direction, their mathematical structure requires a certain care. In some cases, challenging higher-order gradients make these equations hardly tractable. In other cases, these models are based on phase-space formulations that suffer from well-known consistency issues. Here, we present a new complex fluid system that resolves these difficulties. Unlike common approaches, the current system is obtained by applying the fluid closure at the level of the action principle of the original phase-space model. As a result, the system inherits a Hamiltonian structure and retains energy/momentum balance. After discussing some of its structural properties and dynamical invariants, we illustrate the model in the case of pure-dephasing dynamics. We conclude by presenting some invariant planar models.

## Introduction

### Madelung Hydrodynamics in Molecular Motion

The search for cost-effective computational approaches to many-body quantum systems has stimulated a great deal of studies, often exploiting Madelung’s hydrodynamic formulation of quantum mechanics (Madelung 1927). For example, the hydrodynamic approach has led to the development of several trajectory-based algorithms in quantum molecular dynamics (Chattaraj 2011; Garashchuk et al. 2010; Hughes et al. 2009; Kendrick 2003; Wyatt 2005), beyond the well-established Born–Oppenheimer theory. In this setting, one deals with the coupled motion of both nuclei and electrons in a molecular system for which the full Schrödinger equation represents a formidable challenge. The latter is normally tackled by convenient approaches that alleviate the computational complexity. Among these approaches, Madelung hydrodynamics has the great advantage of restoring the concept of trajectory, which can then be exploited to model the nuclear dynamics while the electronic motion is usually approximated as a finite-dimensional quantum system (Agostini et al. 2016; Curchod et al. 2011; Holm et al. 2021).

In this hydrodynamic picture, the Lagrangian trajectories associated to the nuclear motion sweep the electronic unitary evolution. In many important cases (Agostini et al. 2016; Holm et al. 2021), this description is made possible by the observation that any two-particle wavefunction $\Psi ({\varvec{r}},{\varvec{x}},t)$ may be expressed in terms of the *exact factorization* (Abedi et al. 2012), i.e., $\Psi ({\varvec{r}},{\varvec{x}},t)=\chi ({\varvec{r}},t)\psi ({\varvec{x}},t;{\varvec{r}})$, with $|\chi |^2\ne 0$ and $\int |\psi |^2\,\textrm{d}^3x=1$. Here, the semicolon indicates that $\psi$ is a conditional wavefunction so that the coordinate ${\varvec{r}}$ appears merely as a parameter. Notice that this factorization is directly generalizable to the case of two families of several particles, i.e., nuclei with coordinates $\{{\varvec{r}}_j\}$ and electrons with coordinates $\{{\varvec{x}}_k\}$, although here we will deal the simplest possible case of only one nucleus. In addition, to avoid possible complications from functional analysis, we will consider finite-dimensional truncations of the conditional wavefunction. This means that $\psi ({\varvec{x}},t;{\varvec{r}})$ is replaced by a field $\psi (t;{\varvec{r}})$ taking values in the electronic Hilbert space ${\textsf{H}}=\mathbb {C}^n$. Replacing the exact factorization ansatz in the two-particle Schrödinger equation $i\hbar \partial _t\Psi =-\hbar ^2\Delta _{\varvec{r}}\Psi /M+{\widehat{H}}({\varvec{r}})\Psi$ and writing $\chi =\sqrt{D}e^{-iS/\hbar }$, as well as ${\hat{\varrho }}=\psi \psi ^\dagger$, yields the system (Foskett et al. 2019; Holm et al. 2021)

1 $$\begin{aligned} \begin{aligned} M(\partial _t + {\varvec{u}}\cdot \nabla ){\varvec{u}}&= - \nabla V_Q-\langle {\hat{\varrho }}|\nabla {\widehat{H}}\rangle -\frac{\hbar ^2}{2MD}\partial _j\langle D \nabla {\hat{\varrho }}| \partial _j{\hat{\varrho }}\rangle ,\\ i\hbar (\partial _t +{\varvec{u}}\cdot \nabla ){\hat{\varrho }}&= \left[ {\widehat{H}}-\frac{\hbar ^2}{2MD}\textrm{div}(D\nabla {\hat{\varrho }}),{\hat{\varrho }}\right] ,\qquad \partial _t D+\textrm{div}(D{\varvec{u}})=0\,. \end{aligned} \end{aligned}$$

Here, and throughout this paper, $\langle A |B\rangle ={\text {Tr}} (A^\dagger B)$ is the Frobenius inner product on $\mathbb {C}^{n\times n}$ and the Hamiltonian operator ${\widehat{H}}({\varvec{r}})$ is an ${\varvec{r}}$-dependent Hermitian matrix. The latter is usually expressed as ${\widehat{H}}({\varvec{r}})=V({\varvec{r}})\varvec{1}+{\widehat{V}}_I({\varvec{r}})+{\widehat{H}}_Q$, where $V({\varvec{r}})$ is the scalar potential energy, ${\widehat{H}}_Q$ is an ${\varvec{r}}$-independent matrix, and ${\widehat{V}}_I({\varvec{r}})$ is a general interaction term. Also, we have denoted

$$\begin{aligned} {\varvec{u}}:=\frac{1}{M}D\big (\nabla S-\langle \psi |i\hbar \nabla \psi \rangle \big ),\qquad \qquad V_Q:= -\,\frac{\hbar ^2}{2M}\frac{\Delta \sqrt{D}}{\sqrt{D}}. \end{aligned}$$

The density matrix of the electronic system is expressed as ${{\hat{\uprho }}}=\int \Psi ({\varvec{r}})\Psi ^\dagger ({\varvec{r}})\,\textrm{d}^3r=\int D{\hat{\varrho }}\,\textrm{d}^3r$. Equations (1) describe a system in which the quantum hydrodynamic flow sweeps a finite-dimensional quantum system, which also undergoes its own unitary evolution. The latter comprises both the standard term $[{\widehat{H}},{\hat{\varrho }}]$ of von Neumann type and an $\hbar ^2$-term carrying second-order gradients. In turn, these terms also feed back in the hydrodynamic momentum equation. Together with the *quantum potential* $V_Q$, these terms make the level of complexity of this system rather challenging. In particular, the quantum potential in Madelung hydrodynamics has motivated relevant works in PDE analysis (Gamba and Jüngel 2002; Gamba et al. 2009; Degond et al. 2007a, b). We observe that (1) has the structure of a complex fluid model, in which a macroscopic fluid equation is coupled to the evolution of a microscopic component. While in this case the latter is given by the density matrix $\hat{\varrho }$ of the quantum state, in liquid crystal theory, for example, the microscopic component is identified by the *Q*-tensor of the molecular rotational state (de Gennes 1971). In physical terms, the quantum potential $V_Q$ is known to be responsible for the emergence of high-frequency and short-wavelength patterns that may lead to the formation of regions of space where the density *D* vanishes (quantum vortices) (Bialynicki-Birula et al. 2000; Dirac 1931; Foskett and Tronci 2024). Even if one attempted to discard $V_Q$, the other nonlinear terms involving second-order gradients would still lead to analogous complications. Overall, the full hydrodynamic system (1) is accompanied by computational difficulties that still challenge the chemistry community (Garashchuk et al. 2023; Gu and Franco 2017; Suzuki and Watanabe 2016; Zhao and Makri 2003).

### Mixed Quantum–Classical Fluid Models

We observe that the difficulties mentioned above are removed if we deliberately eliminate the higher gradient terms in the first two equations of (1). This step is usually accompanied by arguments resorting to well-established WKB methods and corresponds to a classical treatment of the hydrodynamic flow: indeed, upon recalling $\varvec{u}=D(\nabla S-\langle \psi | i\hbar \nabla \psi \rangle )/M$, one verifies that dropping the higher gradient terms in (1) takes the equation for the phase function $S({\varvec{r}},t)$ into a classical Hamilton–Jacobi equation. In the context of molecular dynamics, for example, this step corresponds to approximating nuclear motion as classical while leaving the electronic dynamics as fully quantum.

In the case of equations (1), neglecting the second-order $\hbar ^2$-terms yields the *Ehrenfest fluid model* for the interaction of a classical system and a quantum system:

2 $$\begin{aligned}{} & {} M(\partial _t + {\varvec{u}}\cdot \nabla ){\varvec{u}}= -\langle {\hat{\varrho }}|\nabla {\widehat{H}}\rangle ,\qquad \nonumber \\{} & {} \quad i\hbar (\partial _t +{\varvec{u}}\cdot \nabla ){\hat{\varrho }} = [{\widehat{H}},{\hat{\varrho }}],\qquad \partial _t D+\textrm{div}(D{\varvec{u}})=0. \end{aligned}$$

This complex fluid model identifies the simplest case of *mixed quantum–classical dynamics*, which generally comprises a vast set of models that have been devised to avoid the complications arising from fully quantum descriptions. While popular in molecular dynamics, similar approaches have also been proposed in solid state physics (Ben Abdallaha et al. 2009; Hurst et al. 2017), spintronics (Petrović et al. 2018), and quantum cosmology (Bojowald and Ding 2021). Despite its mathematical appeal, the Ehrenfest fluid model (2) is insufficient to provide a reliable description of the coupled dynamics (Akimov et al. 2014; Tully 1998). Thus, much of the computational chemistry community has been developing alternative quantum–classical models beyond the Ehrenfest system (Crespo-Otero and Barbatti 2018).

Among the most popular alternatives to the Ehrenfest model, the *surface hopping* algorithm was recently shown to violate Heisenberg’s uncertainty principle, since the quantum density matrix ${{\hat{\uprho }}}$ may become sign-indefinite (Bondarenko and Tempelaar 2023). The same violation of the Heisenberg principle is also allowed by the *quantum–classical Liouville equation* on phase space (Aleksandrov 1981; Boucher and Traschen 1988; Gerasimenko 1982; Kapral 2006). In more generality, certain well-known consistency issues accompany current quantum–classical approaches beyond the Ehrenfest equations (Agostini et al. 2007). On the one hand, since several studies do not seem to be affected by these issues, the existing approaches continue to be regarded as a useful tool. On the other hand, the systematic formulation of quantum–classical models beyond Ehrenfest dynamics remains a subject of current research (Boucher and Traschen 1988; Crespo-Otero and Barbatti 2018; Chruściński et al. 2011; Diósi 2014; Foskett et al. 2019; Hall and Reginatto 2016; Kapral 2006).

While the above models arise as hydrodynamic formulations of multi-particle systems, similar approaches have also appeared in different contexts to study systems involving a genuine complex fluid component. For example, mixed quantum–classical fluid models were proposed in solvation theory to describe the interaction dynamics of macroscopic liquid solvents with quantum solute molecules (Bousquet et al. 2011; Hughes et al. 2012). Since solvation phenomena occur in a wide variety of natural and technological processes, this solute–solvent interaction acquires crucial importance in different areas. In particular, the fluid-molecule coupling involves challenging nonlinear effects at different scales, which are revealed in unprecedented detail by time-resolved ultrafast experiments (Rosspeintner et al. 2013). Current solvation approaches are mostly based on different combinations of computationally expensive atomistic approaches with continuum descriptions involving many substantial approximations (Santoro et al. 2021). A direction that remains little explored is the development of quantum–classical multiscale approaches combining microscopic solute dynamics with a mesoscopic solvent description. A remarkable effort in this direction is found in Bousquet et al. (2011); Burghardt and Bagchi (2006), where the authors formulate a quantum–classical hydrodynamic model by applying the moment method from kinetic theory to the quantum–classical Liouville equation. Depending on the closure adopted for the operator-valued tensor-density ${\widehat{\Pi }}$, the system presented in Bousquet et al. (2011) reads

3 $$\begin{aligned}{} & {} \partial _t\hat{\varvec{g}} = -{\text {div}}{\widehat{\Pi }} -\frac{1}{2}\big ({\tilde{\rho }}(\nabla {\widehat{H}}_{\textrm{eff}}) +(\nabla {\widehat{H}}_{\textrm{eff}}){\tilde{\rho }}\big )+\frac{1}{i\hbar } \big [\hat{\varvec{g}},{\widehat{H}}_{\textrm{eff}}\big ],\nonumber \\{} & {} i\hbar \partial _t {\tilde{\rho }} +\frac{ i\hbar }{M} {\text {div}}\hat{\varvec{g}} = \big [{\widehat{H}}_{\textrm{eff}},{\tilde{\rho }}\big ]. \end{aligned}$$

Here, ${\tilde{\rho }}$ and $\hat{\varvec{g}}$ are operator-valued density and momentum-density, respectively, so that the fluid density and momentum are $D={\text {Tr}}{\tilde{\rho }}$ and ${{\textbf{m}}}={\text {Tr}}\hat{\varvec{g}}$. Also, ${\widehat{H}}_{\textrm{eff}}$ is an effective Hamiltonian carrying nonlocal terms. Benchmarked in Hughes et al. (2012) for certain closure schemes, the system (3) provides a new promising perspective in solvation models.

Nonetheless, the necessity to address the consistency requirements of quantum–classical dynamics motivates us to consider also complementary modeling strategies to tackle problems in molecular dynamics, solvation theory, and other fields. In particular, this paper presents a new class of mixed quantum–classical models lying beyond Ehrenfest dynamics and still satisfying its consistency properties. Similarly to the work in Bousquet et al. (2011), Burghardt and Bagchi (2006), the present complex fluid model is formulated as a closure scheme of a more fundamental mixed quantum–classical approach on phase space. However, instead of applying the closure at the level of the equations of motion, we will operate on the variational principle underlying a recent phase-space formulation that, unlike other available variants, appears to ensure a series of important consistency properties. As a result, the new hydrodynamic system inherits desirable variational and Hamiltonian structures which are analogous to those appearing in the theory of liquid crystals (Gay-Balmaz and Ratiu 2009; Holm 2002; Tronci 2012).

### Quantum–Classical Dynamics on Phase Space

Several mixed quantum–classical models currently used in molecular dynamics are based on the phase-space formulation of classical mechanics (Burghardt and Parlant 2004; Fang et al. 2018; Kapral 2006; Wu et al. 2022). While doubling the dimensionality of classical motion, this approach offers the opportunity for a wider range of possible approximations leading to computational schemes. Over the years, however, these phase-space formulations have not helped to overcome the consistency issues affecting quantum–classical models beyond Ehrenfest dynamics. Until very recently.

Blending Koopman’s Hilbert-space formulation of classical mechanics with van Hove’s unitary representations in symplectic geometry (Bondar et al. 2019; Koopman 1931; van Hove 1951), the present authors proposed a new phase-space model of quantum–classical mechanics that satisfies all the following criteria (Boucher and Traschen 1988) and still retains the geometric underpinnings of both classical and quantum mechanics (Bondar et al. 2019; Gay-Balmaz and Tronci 2022a, b): (1) the classical system is identified by a probability density at all times; (2) the quantum system is identified by a positive-semidefinite density operator ${{\hat{\uprho }}}$ at all times; (3) the model is equivariant under both quantum unitary transformations and classical canonical transformations; (4) in the absence of an interaction potential, the model reduces to uncoupled quantum and classical dynamics; (5) in the presence of an interaction potential, the *quantum purity* $\Vert {\hat{\uprho }}\Vert ^2$ has nontrivial dynamics (decoherence property).

If $f({\varvec{q}},{\varvec{p}},t)$ denotes the classical Liouville density on phase space and $\uppsi (t;{\varvec{q}},{\varvec{p}}) \in {\textsf{H}}$ is the conditional wavefunction representing the quantum dynamics (now depending on the classical phase-space coordinates), the model proposed in Gay-Balmaz and Tronci (2022a, 2022b) reads as follows

4 $$\begin{aligned} {\partial _t f} +{\text {div}}(f\varvec{{{\mathcal {X}}}})=0 ,\qquad i\hbar ({\partial _t {\widehat{P}}} +\varvec{{{\mathcal {X}}}}\cdot \nabla {\widehat{P}})=\big [\,\widehat{\!{\mathscr {H}}},{\widehat{P}}\big ], \end{aligned}$$

with $${\widehat{P}}=\uppsi \uppsi ^\dagger $$,

5 $$\begin{aligned} \varvec{{{\mathcal {X}}}}= \langle {\widehat{P}}|{{\textbf{X}}}_{\widehat{{{\mathcal {H}}}}}\rangle -\frac{\hbar }{2f}\Big ( \big \langle (f\widehat{\varvec{\Sigma }})\cdot \nabla | {{\textbf{X}}}_{{\widehat{{{\mathcal {H}}}}}}\big \rangle -\big \langle {{\textbf{X}}}_{{\widehat{{{\mathcal {H}}}}}}\cdot \nabla | (f\widehat{\varvec{\Sigma }})\big \rangle \Big ), \qquad \quad \widehat{\varvec{\Sigma }}=i[{\widehat{P}},{{\textbf{X}}}_{{\widehat{P}}}] \nonumber \\ \end{aligned}$$

and

6 $$\begin{aligned} \widehat{\!{\mathscr {H}}}= \widehat{{{\mathcal {H}}}}+i\hbar \Big (\big \{{\widehat{P}}, {\widehat{{{\mathcal {H}}}}}\big \}+\big \{\widehat{{{\mathcal {H}}}}, {\widehat{P}}\big \}-\frac{1}{2f}\big [\big \{ f,\widehat{{{\mathcal {H}}}}\big \},{\widehat{P}}\big ] \Big ). \end{aligned}$$

Here, $\{\cdot ,\cdot \}$ is the canonical Poisson bracket, $\widehat{{{\mathcal {H}}}}({\varvec{q}},{\varvec{p}})$ is the Hermitian Hamiltonian operator parameterized by the classical phase-space coordinates, and we have denoted ${{\textbf{X}}}_{{\widehat{A}}}=(\partial _{{\varvec{p}}}{\widehat{A}},-\partial _{{\varvec{q}}}{\widehat{A}})$. The density matrix of the quantum subsystem is expressed as ${{\hat{\uprho }}}=\int f {\widehat{P}}\,\textrm{d}^3q\textrm{d}^3p$. If we discard the $\hbar$-corrections in both the vector field $\varvec{{{\mathcal {X}}}}$ and the Hermitian generator $\,\widehat{\!{\mathscr {H}}}$, the model reduces to a phase-space variant (Zimmermann and Vaníček 2012) of the Ehrenfest fluid equations (2). Indeed, after replacing $\varvec{{{\mathcal {X}}}}=\langle {\widehat{P}}| {{\textbf{X}}}_{\widehat{{{\mathcal {H}}}}}\rangle$ and $\,\widehat{\!{\mathscr {H}}}={\widehat{{{\mathcal {H}}}}}$ in (4), with ${\widehat{{{\mathcal {H}}}}}({\varvec{q}}, {\varvec{p}})=M^{-1}|{\varvec{p}}|^2/2+{\widehat{H}}({\varvec{q}})$, and taking the hydrodynamic moments $(D,MD{\varvec{u}})=\int (1,{\varvec{p}})f\,\textrm{d}^3p$ and $D{\hat{\varrho }}=\int f{\widehat{P}}\,\textrm{d}^3p$, one recovers equations (2) by neglecting pressure effects.

While Eqs. (4)–(6) appear hardly tractable, a direct calculation of ${\text {div}}\!\varvec{{{\mathcal {X}}}}$ reveals that no gradients of order higher than two appear in Eq. (4). In addition, the variational and Hamiltonian structures of this system (Gay-Balmaz and Tronci 2022a, b) reveal the features occurring in quantum–classical coupling. For example, as we will see, Eqs. (4)–(6) lead to the identification of a quantum–classical Poincaré integral invariant which extends the classical quantity $\oint {\varvec{p}}\cdot \textrm{d}{\varvec{q}}$. In more generality, the system (4)–(6) appears to be the first quantum–classical Hamiltonian model beyond Ehrenfest dynamics that satisfies the consistency criteria listed above.

Given the combination of consistency properties and underlying mathematical structure, we consider Eqs. (4)–(6) as a platform for the formulation of simplified closure models that can be used in physically relevant cases. Indeed, the characteristic nature of Eq. (4) offers the possibility of devising trajectory methods, e.g., following (Foskett et al. 2019; Holm et al. 2021; Tronci and Gay-Balmaz 2023). For example, in Bauer et al. (2023) we recently showed how particle schemes based on (4)–(6) succeed in reproducing crucial dynamical features in both quantum and classical sectors, well beyond the Ehrenfest model. However, the model (4)–(6) involves the full classical phase space, which makes the curse of dimensions particularly challenging. In an attempt to alleviate this problem, here we consider the formulation of a fluid closure on the configuration space. This closure represents a quantum–classical alternative to the quantum hydrodynamic approach in (1) and yet retains correlation effects beyond Ehrenfest dynamics.

### Plan of the Paper

The paper proceeds by illustrating the original quantum–classical phase-space model in Sect. 2. After an overview of its derivation, the underlying action principle is presented in Sect. 2.1, along with a discussion of its geometric structure. The Hamiltonian structure and its associated Casimir invariants are found in Sect. 2.2, while Sect. 2.3 deals with the equivariance properties of the model.

The complex fluid model is formulated in Sect. 3. After introducing the relevant fluid variables, the first closure steps are performed in Sect. 3.1 via a restriction to the configuration space of the original paths on phase space. The crucial advance is presented in Sect. 3.2, where we show that the last term in (8) may be projected to the configuration space by resorting to a Poisson bracket structure known as *Nambu bracket*. This process leads to the appearance of two new scalar functions that are transported along the flow, one of which—the *backreaction field*—incorporates the forces excerpted by the quantum system in the classical fluid flow. Then, the explicit form of the fluid equations is presented in Sect. 3.3, where we emphasize that the new model contains only first-order gradients, in contrast to the fully quantum treatment.

A discussion of the relevant properties of the new model is found in Sect. 4. In Sect. 4.1, we discuss the appearance of a non-Abelian gauge connection previously found in the context of molecular dynamics. In particular, this quantity seems to play an important role in the expression of the fluid stress tensor. The new complex fluid model possesses infinite families of dynamical invariants which are presented in Sect. 4.2, along with a discussion of the underlying Hamiltonian structure. Importantly, the Berry connection appears prominently when expressing the quantum dynamics in terms of wavefunctions, thereby allowing the characterization of a *cross-helicity* hydrodynamic invariant. An interesting specialization is presented in Sect. 4.3, where we show that the proposed model succeeds in retaining backreaction forces, otherwise lost in the Ehrenfest treatment. Finally, a class of invariant planar subsystems is illustrated in Sect. 4.4. In this case, we show that restricting to two spatial dimensions allows for larger families of dynamical invariants that may be used for Lyapunov stability studies.

## Geometric Structure of the Phase-Space Model

As anticipated, we will now review the formulation of the quantum–classical model (4)–(6), with special emphasis on its geometric structure (Gay-Balmaz and Tronci 2022a). Before embarking on this discussion, we will start by presenting the theoretical background. In first place, one adopts Koopman’s Hilbert-space formulation of classical mechanics (Koopman 1931): writing $f=|\chi |^2$ in the classical Liouville equation $\partial _t f=\{H,f\}$ yields $i\hbar \partial _t\chi =i\hbar \{H,\chi \}-\varphi \chi$, where $\varphi ({\varvec{q}},{\varvec{p}})$ is an arbitrary function. Then, since the operator $i\hbar \{H,\_\}+\varphi \_$ is self-adjoint, the *Koopman wavefunction* $\chi ({\varvec{q}},{\varvec{p}},t)$ evolves unitarily, just in the same way as quantum Schrödinger wavefunctions. Guided by a series of arguments in representation theory, van Hove fixed the particular phase term $\varphi ={\varvec{p}}\cdot \partial _{\varvec{p}}H-H$ (van Hove 1951), that is the phase-space form of the Lagrangian usually written as ${\varvec{p}}\cdot {\dot{{\varvec{q}}}}-H$. This step makes Koopman’s original formulation compatible with Hamilton–Jacobi theory (Gay-Balmaz and Tronci 2019).

A first quantum–classical model was obtained by writing the *Koopman-van Hove equation* $i\hbar \partial _t\chi =i\hbar \{H,\chi \}-({\varvec{p}}\cdot \partial _{\varvec{p}}H-H)\chi$ for two interacting systems and then quantizing one of them (Bondar et al. 2019; Gay-Balmaz and Tronci 2019). While ensuring a series of important consistency criteria, the resulting *quantum–classical wave equation* $i\hbar \partial _t\Upsilon =i\hbar \{\widehat{{\mathcal {H}}},\Upsilon \}-({\varvec{p}}\cdot \partial _{\varvec{p}}\widehat{{\mathcal {H}}}-\widehat{{\mathcal {H}}})\Upsilon$ generally allows for the classical Liouville density to become unsigned. Here, $\Upsilon \in L^2(\mathbb {R}^6)\otimes {\textsf{H}}$, where we recall that ${\textsf{H}}$ is the quantum Hilbert space. The issue of classical positivity was addressed by resorting to a physical principle proposed by Sudarshan (1976): any model of mixed quantum–classical dynamics should prevent classical phases from having observable effects. In other words, Hamilton–Jacobi functions are not measurable. This principle led the authors to treat the infinite-dimensional group of $S^1$-valued functions on phase space as a gauge group. Indeed, as shown in Gay-Balmaz and Tronci (2022b), applying a $S^1$-symmetry by a suitable closure relation leads to treating classical phases as a *gauge freedom*. This is in direct analogy with the role of unit complex numbers in standard quantum mechanics. While applying the $S^1$-symmetry to the action principle

7 $$\begin{aligned} \delta \int _{t_1}^{t_2}\!\int {\text {Re}}\big \langle \Upsilon \big |i\hbar \partial _t\Upsilon -\{i\hbar \widehat{{\mathcal {H}}}, \Upsilon \}+({\varvec{p}}\cdot \partial _{\varvec{p}}\widehat{{\mathcal {H}}} -\widehat{{\mathcal {H}}})\Upsilon \big \rangle \,\textrm{d}^3q\textrm{d}^3p\,\textrm{d}t=0 \end{aligned}$$

for the quantum–classical wave equation was found to restore positivity of the classical density, this procedure requires resorting to the Lagrangian trajectories underlying classical phase-space motion. This requirement is accommodated by combining the exact factorization method (Abedi et al. 2012) with the Euler–Poincaré variational theory of continuum media (Holm et al. 1998), which lies as the basis for the new treatment. However, the resulting model $i\hbar (\partial _t+\varvec{{{\mathcal {X}}}}\cdot \nabla )\Upsilon +i\hbar ({\text {div}}\varvec{{{\mathcal {X}}}})\Upsilon /2=\,\widehat{\!{\mathscr {H}}}\Upsilon$ (up to phase factors) is highly nonlinear and a real intuition on its structure can only come from investigating its variational formulation. Here, $\varvec{{{\mathcal {X}}}}$ and $\,\widehat{\!{\mathscr {H}}}$ are given in (5)–(6) upon writing $f{\widehat{P}}=\Upsilon \Upsilon ^\dagger$. Also, if we insist on considering only finite-dimensional quantum systems, then $\Upsilon ({\varvec{q}},{\varvec{p}})$ is a Koopman wavefunction taking values in ${\textsf{H}}=\mathbb {C}^n$ and satisfying $\int \Upsilon ^\dagger \Upsilon \textrm{d}^6 z=1$. To avoid dealing with unnecessary phase factors, the remainder of this section considers this model as given in (4)–(6), that is in terms of the quantities *f* and ${\widehat{P}}$.

### Action Principle and Dynamics on Group Orbits

The basis for the model Eqs. (4)–(6) is given by their underlying action principle $\delta \int _{t_1}^{t_2}\!L\,\textrm{d}t=0$ associated to the Euler–Poincaré Lagrangian

8 $$\begin{aligned} L(\varvec{{{\mathcal {X}}}},f,{\hat{\xi }},{\widehat{P}})=\int \!f\big (\varvec{{{\mathcal {A}}}}\cdot \varvec{{{\mathcal {X}}}}+\big \langle {{\widehat{P}}},i\hbar {\hat{\xi }}-{\widehat{{{\mathcal {H}}}}}-i\hbar \{{{\widehat{P}}},\widehat{{{\mathcal {H}}}}\}\big \rangle \big )\,\textrm{d}^6z . \end{aligned}$$

We refer to Sect. 5.1 in Gay-Balmaz and Tronci (2022b) for the derivation of this Lagrangian from (7) and the corresponding Euler–Poincaré variations. Here, we have denoted ${{\textbf{z}}}=({\varvec{q}},{\varvec{p}})$ and $\langle ,\rangle ={\text {Re}}\langle \,|\,\rangle$, while ${\varvec{{{\mathcal {A}}}}=({\varvec{p}},0)}$ is the coordinate representation of the Liouville one-form ${{{\mathcal {A}}}}={\varvec{{{\mathcal {A}}}}\cdot \textrm{d}{{\textbf{z}}}}={\varvec{p}}\cdot \textrm{d}{\varvec{q}}$, so that $\omega =-\textrm{d}{{{\mathcal {A}}}}$ is the canonical symplectic form. Also, ${\hat{\xi }}({{\textbf{z}}},t)$ is a skew-Hermitian operator and, in the notation of Sect. 1.3, ${{ {\widehat{P}}}({{\textbf{z}}},t):=\uppsi ({{\textbf{z}}},t)\uppsi ({{\textbf{z}}},t)^\dagger }$. As usual in Euler–Poincaré variational theories, the variations $\delta f$ and $\delta \varvec{{{\mathcal {X}}}}$ are constrained so that

9 $$\begin{aligned} \delta f=-{\text {div}}(f\varvec{{{\mathcal {Y}}}}),\qquad \delta \varvec{{{\mathcal {X}}}}=\partial _t\varvec{{{\mathcal {Y}}}} +\varvec{{{\mathcal {X}}}}\cdot \nabla \varvec{{{\mathcal {Y}}}} -\varvec{{{\mathcal {Y}}}}\cdot \nabla \varvec{{{\mathcal {X}}}}, \end{aligned}$$

where $\varvec{{{\mathcal {Y}}}}$ is arbitrary. Analogously, we have

10 $$\begin{aligned} \delta { {\widehat{P}}} = [{{\hat{\zeta }}}, { {\widehat{P}}}] - \varvec{{{\mathcal {Y}}}}\cdot \nabla {\widehat{P}},\qquad \qquad \ \delta {\hat{\xi }} = \partial _t {{{\hat{\zeta }}}} + [ {{{\hat{\zeta }}}}, {\hat{\xi }} ] + \varvec{{{\mathcal {X}}}} \cdot \nabla {{{\hat{\zeta }}}} - \varvec{{{\mathcal {Y}}}}\cdot \nabla {\hat{\xi }}, \end{aligned}$$

where ${{\hat{\zeta }}}$ is an arbitrary skew-Hermitian operator. These variations arise by standard Euler–Poincaré reduction from Lagrangian to Eulerian variables (Holm et al. 1998). In particular, if $\varvec{\eta }({{\textbf{z}}}_0,t)$ is the diffeomorphic Lagrangian path on phase space and $U({{\textbf{z}}},t)$ is a unitary operator, we have the following relations between Lagrangian and Eulerian variables:

11 $$\begin{aligned} f=\varvec{\eta }_*f_0,\qquad {{\widehat{P}}}=(U{ {\widehat{P}}}_0U^\dagger )\circ \varvec{\eta }^{-1},\quad \dot{\varvec{\eta }}=\varvec{{{\mathcal {X}}}}\circ \varvec{\eta },\quad {\hat{\xi }}={\dot{U}}U^\dagger \circ \varvec{\eta }^{-1}, \end{aligned}$$

where $f_0$ and ${\widehat{P}}_0$ are initial conditions. Here, ${\varvec{\eta }_*f_0= (f_0/\det \nabla \varvec{\eta }) \circ \varvec{\eta }^{-1}}$ denotes the push-forward of the initial density by $\varvec{\eta }$. We will denote the space of densities on the phase space $T^*Q$ by ${\text {Den}}(T^*Q)$, which is identified with the distributional dual ${{{\mathcal {F}}}}(T^*Q)^*$ of scalar functions ${{{\mathcal {F}}}}(T^*Q)$ via the $L^2$-pairing. Also, we have ${\widehat{P}}\in {{{\mathcal {F}}}}(T^*Q,{\text {Her}}({\textsf{H}}))$, where ${\text {Her}}({\textsf{H}})$ is the space of Hermitian operators on ${\textsf{H}}$ and ${{{\mathcal {F}}}}(T^*Q,{\text {Her}}({\textsf{H}}))$ denotes the space of mappings $T^*Q\rightarrow {\text {Her}}({\textsf{H}})$. While *f* is positive-definite, ${\widehat{P}}$ is only positive-semidefinite, and we will regard these properties as being preserved in time from suitable initial conditions. The first two relations in (11) indicate that the evolution of

$$\begin{aligned} (f,{ {\widehat{P}}})\in {\text {Den}}(T^*Q)\times {{{\mathcal {F}}}} (T^*Q,{\text {Her}}({\textsf{H}})) \end{aligned}$$

occurs on orbits of the semidirect-product group

$$\begin{aligned} {\text {Diff}}(T^*Q)\,\circledS \, {{{\mathcal {F}}}}(T^*Q,{{{\mathcal {U}}}}({\textsf{H}})), \end{aligned}$$

where ${\text {Diff}}(T^*Q)$ is the group of phase-space diffeomorphisms, and ${{{\mathcal {F}}}}(T^*Q,{{{\mathcal {U}}}}({\textsf{H}}))$ denotes the space of functions on $T^*Q$ taking values into the group ${{{\mathcal {U}}}}({\textsf{H}})$ of unitary operators on the quantum Hilbert space ${\textsf{H}}$. In particular, these orbits are determined by the group action identified by the first two in (11), that is the composition of the standard conjugation representation of ${{{\mathcal {F}}}}(T^*Q,{{{\mathcal {U}}}}({\textsf{H}}))$ and the push-forward action of ${\text {Diff}}(T^*Q)$. This evolution on group orbits will be crucial in later sections. In this paper, we will consider $Q=\mathbb {R}^3$ for simplicity, although the treatment extends naturally to a smooth manifold.

This geometric structure reflects directly in Eqs. (4)–(6). Indeed, taking the time derivative of the first two relations in (11) yields $\partial _t f+{\text {div}}(f\varvec{{{\mathcal {X}}}})=0$ and ${\partial _t{ {\widehat{P}}}+\varvec{{{\mathcal {X}}}}\cdot \nabla { {\widehat{P}}}=[{\hat{\xi }},{ {\widehat{P}}}]}$, respectively, and we observe the appearance of Lie-transport operators. Furthermore, upon taking variations of (8), the action principle $\delta \int _{t_1}^{t_2}L\,\textrm{d}t=0$ yields

12 $$\begin{aligned}{} & {} \varvec{{{{\mathcal {X}}}}}={{\textbf{X}}}_{\textstyle \frac{\delta h}{ \delta f}}-\frac{1}{f}\bigg \langle {\frac{\delta h}{\delta { {\widehat{P}}}}}\Big | {{\textbf{X}}}_{{\widehat{P}}}\bigg \rangle ,\quad \ \ \bigg [i\hbar {\hat{\xi }}-\frac{1}{f}\frac{\delta h}{ \delta { {\widehat{P}}}},{ {\widehat{P}}}\bigg ]=0, \nonumber \\{} & {} \quad \text {where}\quad h=\!\int \!f\big \langle {\widehat{{{\mathcal {H}}}}}+i\hbar \{{ {\widehat{P}}},\widehat{{{\mathcal {H}}}}\}\big \rangle \,\textrm{d}^6z \end{aligned}$$

and we have used $\langle A\rangle :=\langle { {\widehat{P}}},A\rangle$. For later purpose, we recall the definition of the real-valued pairing that is $\langle A,B\rangle = {\text {Re}} \langle A | B \rangle$. Then, after various manipulations we recover the system (4)–(6). The purely quantum and classical cases are found by restricting to the cases ${{\textbf{X}}}_{\widehat{{{\mathcal {H}}}}}=0$ and $\widehat{{{\mathcal {H}}}}={{{\mathcal {H}}}}\varvec{1}$, respectively (Gay-Balmaz and Tronci 2022a). We readily realize that it would be rather unwise to try to obtain some information on this model by looking at the explicit Eqs. (4)–(6) obtained by expanding the functional derivatives ${\delta h}/{\delta f}$ and ${\delta h}/{\delta { {\widehat{P}}}}$. Instead, we are motivated to accept the guidance of their underlying geometric structure. While we have seen how the latter manifests in the variational formulation (8)–(10), we now consider the corresponding Hamiltonian setting.

### Hamiltonian Structure and Poincaré Invariant

As we will see below, the Hamiltonian structure of the model (4)–(6) is rather peculiar and we are not aware of similar structures occurring elsewhere in continuum mechanics.

First, it is convenient to introduce the weighted variable $\mathcal{{\widehat{P}}}=f {\widehat{P}}$ so that $f={\text {Tr}}\mathcal{{\widehat{P}}}$ and $\langle A\rangle =\langle \mathcal{{\widehat{P}}},A\rangle /{\text {Tr}}\mathcal{{\widehat{P}}}$. Then, the Euler–Poincaré Lagrangian (8) may be expressed entirely in terms of the variables $(\varvec{{{\mathcal {X}}}},{\hat{\xi }},\mathcal{{\widehat{P}}})$. Going through the same steps as above leads to rewriting (12) as $\varvec{{{{\mathcal {X}}}}}=\langle {{\textbf{X}}}_{{\delta h}/{\delta \mathcal{{\widehat{P}}}}}\rangle$ and ${[i\hbar {\hat{\xi }}-{\delta h}/{ \delta \mathcal{{\widehat{P}}}},\mathcal{{\widehat{P}}}]=0}$, where ${ h=\int \big \langle {\widehat{{{\mathcal {H}}}}}{\text {Tr}}\mathcal{{\widehat{P}}}+i\hbar \{\mathcal{{\widehat{P}}},\widehat{{{\mathcal {H}}}}\}\big \rangle \,\textrm{d}^6z}$. Then, the Hamiltonian equation

13 $$\begin{aligned} i\hbar \frac{\partial \mathcal {\widehat{P}}}{\partial t} +i\hbar {\text {div}}\!\bigg (\mathcal{{\widehat{P}}}\Big \langle {{\textbf{X}}}_{\textstyle \frac{\delta h}{\delta \mathcal{{\widehat{P}}}}}\Big \rangle \bigg )=\bigg [\frac{\delta h}{\delta \mathcal{{\widehat{P}}}},\mathcal{{\widehat{P}}}\bigg ] \end{aligned}$$

leads directly to the following bracket structure via the usual relation ${\dot{f}}=\{\!\!\{f,h\}\!\!\}$:

14 $$\begin{aligned} \{\!\!\{k,h\}\!\!\}(\mathcal {{\widehat{P}}})=\int \!\frac{1}{{\text {Tr}}\mathcal {{\widehat{P}}} }\bigg (\mathcal {{\widehat{P}}}: \bigg \{\frac{\delta k}{\delta \mathcal {{\widehat{P}}}},\frac{\delta h}{\delta \mathcal {{\widehat{P}}}}\bigg \}: \mathcal {{\widehat{P}}} \bigg )\textrm{d}^6z - \int \!\left\langle \mathcal {{\widehat{P}}},\frac{i}{\hbar }\!\left[ \frac{\delta k}{\delta \mathcal {{\widehat{P}}}},\frac{\delta h}{\delta \mathcal {{\widehat{P}}}}\right] \right\rangle \textrm{d}^6z,\nonumber \\ \end{aligned}$$

where we have introduced the convenient notation ${A:B}={\text {Tr}}(AB)$. The proof that the bracket (14) is Poisson involves a combination of results in Lagrangian and Poisson reduction (Gay-Balmaz and Tronci 2022b). On the one hand, we observe that the second term is naturally inherited from the usual Lie–Poisson structure underlying the quantum Liouville equation. On the other hand, the first term is a new type of Poisson bracket which reduces to the Lie–Poisson structure for the classical Liouville equation for $f={\text {Tr}}\mathcal{{\widehat{P}}}$ if both functional derivatives are a multiple of the identity.

Equation (13) easily leads to characterizing the Casimir invariant $C_1={\text {Tr}}\!\int \! f\Phi (\mathcal{{\widehat{P}}}/f)\,\textrm{d}^6z$ for any matrix analytic function $\Phi$. Also, upon writing $\mathcal{{\widehat{P}}}=f\uppsi \uppsi ^\dagger$, one finds the quantum–classical Poincaré integral invariant: $\oint _{\varvec{c}(t)}({\varvec{p}}\cdot \textrm{d}{\varvec{q}}+\langle \uppsi ,i\hbar \textrm{d}\uppsi \rangle ) =const.$ for any loop $\varvec{c}(t)=\varvec{\eta }(\varvec{c}_0,t)$ in phase space, where we recall that $\varvec{\eta }(t)$ is the flow of $\varvec{{\mathcal {X}}}$. Then, by Stokes theorem, one identifies a Lie-transported quantum–classical two-form on $T^*Q$, that is $\Omega (t)=\varvec{\eta }_*\Omega (0)$, with $\Omega (t):=\omega +\hbar {\text {Im}}{\langle \textrm{d}\uppsi (t)|\wedge \textrm{d}\uppsi (t)\rangle }$, so that $\Omega (t)$ is symplectic if it is so initially. As a result, one finds the additional class of Casimir invariants $C_2=\int f\Theta \big (f^{-1}\Omega \wedge \Omega \wedge \Omega \big )\textrm{d}^6z$, where $\Omega \wedge \Omega \wedge \Omega$ is a volume form and $\Theta$ is any scalar function of one variable. These Casimirs may be used to construct quantum–classical extensions of Gibbs/von Neumann entropies (Gay-Balmaz and Tronci 2022a).

### Quantum–Classical von Neumann Operator

We observe that the Hamiltonian energy functional *h* in (12) is not simply given by the usual average of the Hamiltonian operator $\widehat{{{\mathcal {H}}}}$. Indeed, the $\hbar$-term seems to play a crucial role in taking the model (4)–(6) beyond simple Ehrenfest dynamics. As discussed in Gay-Balmaz and Tronci (2022a), this suggests that the quantum–classical correlations trigger extra energy terms that are not usually considered. A similar situation occurs in standard quantum mechanics with spin-orbit coupling, that is the coupling of the quantum spin to the force acting on the orbital degrees of freedom. See Remark 4.1 below.

One may still rearrange the last relation in (12) in such a way that the total energy is formally given by an average of $\widehat{{{\mathcal {H}}}}$. Following this route leads to rewriting the last relation in (12) as $h={\text {Tr}}\int \!\widehat{{{\mathcal {D}}}}\widehat{{{\mathcal {H}}}}\,\textrm{d}^6z$, where

15 $$\begin{aligned} \widehat{{{\mathcal {D}}}} = f{{\widehat{P}}}+\frac{i\hbar }{2}{\text {div}}\!\big (f[{\widehat{P}}, {{\textbf{X}}}_{{\widehat{P}}}]\big ) \end{aligned}$$

is a measure-valued von Neumann operator. Then, classical and quantum densities are simply given by taking the trace and integral of $\widehat{{{\mathcal {D}}}}$, respectively. Unlike the quantum density operator, we observe that the hybrid operator $\widehat{{{\mathcal {D}}}}$ is not sign-definite. Remarkably, however, $\widehat{{{\mathcal {D}}}}$ enjoys the equivariance properties

16 $$\begin{aligned} \widehat{{{\mathcal {D}}}}(\tilde{\varvec{\eta }}_*f, \tilde{\varvec{\eta }}_*{\widehat{P}})=\tilde{\varvec{\eta }}_* \widehat{{{\mathcal {D}}}}(f,{\widehat{P}}) \qquad \text { and }\qquad \widehat{{{\mathcal {D}}}}(f,{\mathscr {U}}{\widehat{P}} {\mathscr {U}}^\dagger )={\mathscr {U}}\widehat{{{\mathcal { D}}}}(f,{\widehat{P}}){\mathscr {U}}^\dagger , \end{aligned}$$

where $\tilde{\varvec{\eta }}$ is a symplectic diffeomorphism on $T^*Q$ and ${\mathscr {U}}\in {{{\mathcal {U}}}}({\textsf{H}})$. These two properties ensure the following dynamics in the classical and quantum sector, respectively (Gay-Balmaz and Tronci 2022b): ${\partial _t f}={\text {Tr}}\{\widehat{{{\mathcal {H}}}},\widehat{{{\mathcal {D}}}}\}$ and $i\hbar {\textrm{d}{{\hat{\uprho }}}}/{\textrm{d}t}=\int [\widehat{{{\mathcal {H}}}},\widehat{{{\mathcal {D}}}}]\,\textrm{d}^6z$. For example, upon denoting $\widehat{\varvec{\Sigma }}=i[{\widehat{P}},{{\textbf{X}}}_{{\widehat{P}}}]$, the first property is verified directly by writing

$$\begin{aligned}{} & {} {\varvec{{{\mathcal {X}}}}} =f^{-1}\langle \widehat{{{\mathcal {D}}}}, {{\textbf{X}}}_ {\widehat{{{\mathcal {H}}}}}\rangle +\hbar f^{-1}{\text {div}}\!\big (f{\text {Tr}}( {{{\textbf{X}}}_ {\widehat{{{\mathcal {H}}}}}\wedge \widehat{\varvec{\Sigma }}})\big ),\\{} & {} \quad \text { where }\quad ( {{\textbf{X}}}_ {\widehat{{{\mathcal {H}}}}}\wedge \widehat{\varvec{\Sigma }})^{jk}:=\frac{1}{2}\big ( X_ {\widehat{{{\mathcal {H}}}}\,}^j{{\widehat{\Sigma }}}^k-{{\widehat{\Sigma }}}^j X_ {\widehat{{{\mathcal {H}}}}}^k\big ) \end{aligned}$$

identifies a bivector. With this expression of $\varvec{{{\mathcal {X}}}}$, one recognizes that ${\text {div}}\!\varvec{{{\mathcal {X}}}}={\text {Tr}}\{{\widehat{P}}+\hbar {\text {div}}\widehat{\varvec{\Sigma }},\widehat{{{\mathcal {H}}}}\}/2$ $-\hbar {\text {div}}{\text {Tr}}(\{\log f,\widehat{{{\mathcal {H}}}}\}\widehat{\varvec{\Sigma }})/2$ involves only first- and second-order gradients. In more generality, due to the equivariance properties (16), the hybrid von Neumann operator acquires an important role in different aspects, such as the quantum–classical momentum balance (Gay-Balmaz and Tronci 2022a).

## Formulation of the Fluid Closure

Given the level of complexity of the model (4)–(6), it is desirable to develop suitable closure schemes that can substantially simplify the treatment. In particular, here we present a fluid closure in which the classical system is represented by a fluid with density and momentum

17 $$\begin{aligned} {D}({\varvec{q}})=\int \! f({\varvec{q}},{\varvec{p}})\,\textrm{d}^3 p,\qquad \qquad {{\textbf{m}}}({\varvec{q}})=\int \! {\varvec{p}}f({\varvec{q}},{\varvec{p}})\,\textrm{d}^3 p , \end{aligned}$$

respectively, while the measure-valued density $\mathcal{{\widehat{P}}}({\varvec{q}},{\varvec{p}}) = f(\varvec{q},\varvec{p}) \widehat{P}(\varvec{q},\varvec{p})$ on phase space is replaced by

18 $$\begin{aligned} \int \!f({\varvec{q}},{\varvec{p}}){\widehat{P}}({\varvec{q}},{\varvec{p}})\,\textrm{d}^3 p=:{D}({\varvec{q}}){{\hat{\varrho }}}({\varvec{q}}) , \end{aligned}$$

where ${{\text {Tr}}{{\hat{\varrho }}}({\varvec{q}})=1}$. In particular, in this work we focus on quantum–classical Hamiltonians of the type

19 $$\begin{aligned} \widehat{{{\mathcal {H}}}}({\varvec{q}},{\varvec{p}})=\frac{1}{2M}|{\varvec{p}}|^2+{\widehat{H}}({\varvec{q}}). \end{aligned}$$

While we could implement the moment method directly on the equations of motion, as customary in the kinetic theory of gases, here we perform the fluid closure at the level of the variational structure (8)–(10) underlying the full phase-space model. Before proceeding, we emphasize that the current treatment does not include dissipation and diffusion. Indeed, we rely on the possibility to add standard diffusion and dissipation terms *a posteriori*, once the conservative equations are written explicitly. Similarly, pressure effects arising from the purely classical kinematic term ${\varvec{p}}\cdot \partial _{{\varvec{q}}} f/M$ in the first equation of (4) will also be added *a posteriori* by resorting to a conventional equation of state.

### Restriction to Cotangent Lifts

So far the classical motion has been identified with diffeomorphic paths $\varvec{\eta }$ on phase space via their generating vector field $\varvec{{{\mathcal {X}}}}$ defined in (11). Each of these paths acts on the classical density as well as on the unitary operators governing the quantum dynamics as in (11). In order to obtain a fluid description of the classical motion, the latter needs to be restricted to occur on the configuration space. Since we want to operate on the Eulerian action principle associated to the Lagrangian (8), we need to restrict the phase-space vector field $\varvec{{{\mathcal {X}}}}$ to generate only transformations on the configuration space *Q*. We also ask for these transformations to possess a natural extension to phase space. Such a class of transformations is well known under the name of *cotangent lifts* (or simply *lifts*) of diffeomorphisms. In particular, any diffeomorphism ${\varvec{\upeta }}\in {\text {Diff}}(Q)$ induces a phase-space diffeomorphism ${\varvec{\upeta }}^{\textsf{L}}\in {\text {Diff}}(T^*Q)$, that is the ‘lift’ of ${\varvec{\upeta }}$, which reads ${\varvec{\upeta }}^\textsf{L}({\varvec{q}},{\varvec{p}})=\big ({\varvec{\upeta }}({\varvec{q}}),\nabla {\varvec{\upeta }} ^{-1} (\varvec{\upeta }({\varvec{q}}))\cdot {\varvec{p}}\big )$. Notice that we have $\varvec{\upeta }^{\textsf{L}}_*{{{\mathcal {A}}}}={{{\mathcal {A}}}}$, where ${{{\mathcal {A}}}}={\varvec{p}}\cdot \textrm{d}{\varvec{q}}$. Likewise, in terms of Eulerian variables, any vector field ${\varvec{u}}({\varvec{q}})$ identifies a lift on phase space, which reads ${\varvec{u}}^\textsf{L}({\varvec{q}},{\varvec{p}})=\big ({\varvec{u}}({\varvec{q}}),-{\nabla {\varvec{u}}({\varvec{q}})\cdot {\varvec{p}}}\big )$. Thus, as a first step, we will restrict phase-space paths to cotangent lifts of paths on the configuration space, and this amounts to replacing

$$\begin{aligned} \int \!f({\varvec{q}},{\varvec{p}}) \varvec{{{\mathcal {A}}}}({\varvec{q}},{\varvec{p}})\cdot \varvec{{{\mathcal {X}}}}({\varvec{q}},{\varvec{p}})\,\textrm{d}^6z \ \longrightarrow \, \int {{\textbf{m}}}({\varvec{q}})\cdot {\varvec{u}}({\varvec{q}})\,\textrm{d}^3q \end{aligned}$$

in (8). Here, we have used the second definition in (17) while the vector field ${\varvec{u}}$ is defined in terms of Lagrangian paths as $\dot{{\varvec{\upeta }}}={\varvec{u}}\circ {\varvec{\upeta }}$. Notice that, on phase space, one also has $\dot{{\varvec{\upeta }}}^\textsf{L}={\varvec{u}}^{\textsf{L}}\circ {\varvec{\upeta }}^{\textsf{L}}$.

In pursuing our restriction to subgroup transformations on the configuration space, we also need to deal with the quantum propagators $U\in {{{\mathcal {F}}}}(T^*Q,{{{\mathcal {U}}}}({\textsf{H}}))$, which are again mappings defined on the classical phase space. In order to restrict to the configuration space, here we will consider only transformations ${\textsf{U}}({\varvec{q}})$ in the subgroup ${{{\mathcal {F}}}}(Q,{{{\mathcal {U}}}}({\textsf{H}}))$. Then, the quantum generator of motion ${\hat{\xi }}$ in (11) is replaced by ${\hat{\upxi }}=\dot{\textsf{U}}\textsf{U}^{-1}\circ {\varvec{\upeta }}^{-1}$, with ${\textsf{U}}\in {{{\mathcal {F}}}}(Q,{{{\mathcal {U}}}}({\textsf{H}}))$ and ${\varvec{\upeta }}\in {\text {Diff}}(Q)$. Consequently, by recalling (18), we make the following replacement in (8):

$$\begin{aligned} \int \!f({\varvec{q}},{\varvec{p}})\big \langle { {\widehat{P}}({\varvec{q}},{\varvec{p}})},i\hbar {\hat{\xi }}({\varvec{q}},{\varvec{p}})\big \rangle \,\textrm{d}^6z \ \longrightarrow \,\int \!D({\varvec{q}})\big \langle { {\hat{\varrho }}({\varvec{q}})},i\hbar {\hat{\upxi }}({\varvec{q}})\big \rangle \,\textrm{d}^3q. \end{aligned}$$

Another important step concerns the term $\int \!f\langle { {\widehat{P}}},\widehat{{{\mathcal {H}}}}\rangle \,\textrm{d}^6z$ in (8). At this stage, we adopt the *cold-fluid* closure $f( {\varvec{q}},{\varvec{p}})=D( {\varvec{q}})\delta ({\varvec{p}}-{{\textbf{m}}}({\varvec{q}})/D({\varvec{q}}))$, thereby discarding pressure effects. Upon using (19), this expression of the phase-space density leads to the further replacement

$$\begin{aligned} \int \!f\langle {\widehat{P}},\widehat{{{\mathcal {H}}}}\rangle \,\textrm{d}^6z \ \longrightarrow \, \int \Big (\frac{|{{\textbf{m}}}|^2}{2MD}+D\langle { {\hat{\varrho }}},{\widehat{H}}\rangle \Big )\textrm{d}^3q. \end{aligned}$$

Notice that the cold-fluid closure is used here only as an intermediate step restricting to the simplest possible case. We will restore pressure effects later on by adding a fluid internal energy term to the right of the relation above.

### The Quantum Backreaction

So far, the quantities in the Lagrangian (8) were restricted to the configuration space, except for the last term. The latter is responsible for the *quantum backreaction* on the classical flow beyond the usual Hellman–Feynman force averages (Feynman 1939) already appearing on the right-hand side of the momentum equation in the Ehrenfest model (2). The last term in (8) is responsible for a much intricate structure of Eqs. (4)–(6), which involve gradients of the quantum variable ${\widehat{P}}$ and make the identification of a closure far from obvious. Here, we will proceed in stages and pursue the simplest possible closure method to retain nontrivial backreaction effects. Similarly to the procedure in the previous section, here we will continue to operate on the Lagrangian (8), thereby avoiding the necessity to control the various gradients in (4)–(6).

We start by rearranging the last term in (8) as

20 $$\begin{aligned} \int \!f\big \langle { {\widehat{P}}},i\hbar \{{ {\widehat{P}}},\widehat{{{\mathcal {H}}}}\}\big \rangle \,\textrm{d}^6z&= \frac{1}{2}\int \!\big \langle {\widehat{{{\mathcal {H}}}}}, i\hbar {\text {div}}[{ {\widehat{P}}},f{{\textbf{X}}}_{ {\widehat{P}}}] \big \rangle \,\textrm{d}^6z\nonumber \\&= \frac{1}{2}\int \!\bigg \langle {{\widehat{H}}},i\hbar {\text {div}}\!\int [{\widehat{P}},f\partial _{\varvec{p}}{ {\widehat{P}}}]\,\textrm{d}^3 p\bigg \rangle \,\textrm{d}^3q\,, \end{aligned}$$

where the second divergence involves only position coordinates in configuration space. The first equality above follows from a slight rearrangement involving the projection of $i \{{ {\widehat{P}}},\widehat{{{\mathcal {H}}}}\}=i\partial _k {\widehat{P}}X_{\widehat{{{\mathcal {H}}}}}^k$ on its Hermitian part as well as integration by parts. Also, the second equality follows from (19). Upon resorting to the cold-fluid closure, we recall (18) to write ${\hat{\varrho }}={{\widehat{P}}}|_{{\varvec{p}}={{\textbf{m}}}/D}$, and eventually perform the replacement

$$\begin{aligned} {\text {div}}\!\int [{\widehat{P}},f\partial _{\varvec{p}}{ {\widehat{P}}}]\,\textrm{d}^3 p \ \longrightarrow \ {\text {div}}[{\hat{\varrho }},\widehat{\varvec{\kappa }}] ,\qquad \text { with }\qquad \widehat{\varvec{\kappa }}= D\partial _{\varvec{p}}{ {\widehat{P}}}|_{{\varvec{p}}={{\textbf{m}}}/D}. \end{aligned}$$

At this point, our closure problem amounts to finding a closure for the operator-valued current $\widehat{\varvec{\kappa }}$. In particular, we need to find a suitable expression of $\widehat{\varvec{\kappa }}$ in terms of ${\hat{\varrho }}$ and $\nabla {\hat{\varrho }}$. For the sake of simplicity, here we assume a linear relation between $\widehat{\varvec{\kappa }}$ and $\nabla {\hat{\varrho }}$.

To proceed further, we will be guided by the properties of the original model (4)–(6). For example, we notice that the term ${\text {div}} [{\widehat{P}},f{{\textbf{X}}}_{ {\widehat{P}}}]= f[\partial _k {\widehat{P}},X^k_{ {\widehat{P}}}]+[{\widehat{P}},X^k_{ {\widehat{P}}}]\partial _kf$ in the first line of (20) does not produce gradients of order higher than one. Then, in order to avoid the emergence of higher-order gradients in ${\text {div}}[{\hat{\varrho }},\widehat{\varvec{\kappa }}]= [\partial _k{\hat{\varrho }},{\widehat{\kappa }}^k]+ [{\hat{\varrho }},{\text {div}}\widehat{\varvec{\kappa }}]$, the assumption of linearity between $\widehat{\varvec{\kappa }}$ and $\nabla {\hat{\varrho }}$ leaves us with the choice

$$\begin{aligned} \widehat{\varvec{\kappa }}=-\varvec{\beta }\times \nabla {\hat{\varrho }} , \end{aligned}$$

where $\varvec{\beta }({\varvec{q}},t)\cdot \textrm{d}{\varvec{q}}$ is a real-valued differential one-form and the minus sign is chosen for later convenience. Notice that ${{\text {Tr}}{\hat{\varrho }}=1\implies {{\text {Tr}}\widehat{\varvec{\kappa }}=0}}$ and, in the case ${{{\hat{\varrho }}}=\psi \psi ^\dagger }$, we also have $\langle {\hat{\varrho }}|\widehat{\varvec{\kappa }}\rangle =0$. While this possible closure will be explored elsewhere, here we deal with the case $\varvec{\beta }=c\nabla b$ for some scalar functions $b({\varvec{q}},t)$ and $c({\varvec{q}},t)$, that is $\widehat{\varvec{\kappa }}=c\nabla {\hat{\varrho }}\times \nabla b$.

With this expression of $\widehat{\varvec{\kappa }}$, the Lagrangian (8) finally reduces to

21 $$\begin{aligned}{} & {} l({{\textbf{m}}},\varvec{u},D,{\hat{\upxi }},{\hat{\varrho }},b,c)\nonumber \\{} & {} \quad =\int \!\Big ({{\textbf{m}}}\cdot \varvec{u}-\frac{|{{\textbf{m}}}|^2}{2MD}+\big \langle {\hat{\varrho }},i\hbar D{\hat{\upxi }}-D{{\widehat{H}}}-i\hbar c\{{ {\hat{\varrho }}},{\widehat{H}}\}_b\big \rangle \Big )\textrm{d}^3q , \end{aligned}$$

where

22 $$\begin{aligned} \{F,G\}_b:=\nabla b\cdot \nabla F\times \nabla G \end{aligned}$$

defines a Poisson bracket structure of Nambu type (Nambu 1973) on any functions *F* and *G* and for any function *b*. Since the latter is responsible for the backreaction terms in the new Lagrangian (21), we will refer to *b* as the *backreaction field*. We observe the striking analogy between the last term in (21) and the last term in (8). For later purpose, we notice that $\langle {\hat{\varrho }},i\hbar \{{ {\hat{\varrho }}},{\widehat{H}}\}_b\rangle =\langle {\hat{\varrho }},i\hbar [\nabla { {\hat{\varrho }}},\nabla b\times \nabla {\widehat{H}}]\rangle /2$.

#### Remark 3.1

(*Extension to higher dimensions*) The present treatment can be extended to consider the entire coordinate space $\mathbb {R}^{3d}$ for a molecule with *d* nuclei. In this case, each term in the Lagrangian (21) would be replaced by its natural higher-dimensional generalization. In particular, the last term would now involve the direct sum of the same Nambu bracket *d* times. More explicitly, the Nambu bracket (22) would be replaced by the more general Poisson bracket $\{F,G\}_b({\varvec{r}}_1,\ldots ,{\varvec{r}}_d)=\sum _{k=1}^d\nabla _{\!{\varvec{r}}_k} b\cdot \nabla _{\!{\varvec{r}}_k} F\times \nabla _{\!{\varvec{r}}_k} G$.

Besides *b* and *c*, which are dealt with below, the Lagrangian (21) involves the following quantities:

23 $$\begin{aligned} D:=\upeta _*D_0,\quad {{\hat{\varrho }}}:=({\textsf{U}}{ {\hat{\varrho }}}_0{\textsf{U}}^\dagger ) \circ \varvec{\upeta } ^{-1}, \varvec{u}:= \dot{\varvec{\upeta }}\circ \varvec{\upeta } ^{-1},\quad {\hat{\upxi }}:=\dot{\textsf{U}}\textsf{U}^\dagger \circ \varvec{\upeta }^{-1}. \end{aligned}$$

Here, the last two expressions arise from the restrictions performed in Sect. 3.1, while the first two are obtained by combining the definitions in (17)–(18) with the first two relations in (11), as well as replacing $\varvec{\eta }\rightarrow \varvec{\upeta }$ and ${\hat{\xi }}\rightarrow {\hat{\upxi }}$. In particular, the first two in (23) yield the auxiliary equations

24 $$\begin{aligned} \partial _t D+{\text {div}}(D\varvec{u})=0,\qquad \partial _t {{\hat{\varrho }}}+\varvec{u}\cdot {{\hat{\varrho }}} =[{\hat{\upxi }},{{\hat{\varrho }}}], \end{aligned}$$

which accompany the variational principle associated to (21).

The scalar field *c* is inserted in the expression of $\widehat{\varvec{\kappa }}$ in such a way that the last integral in (21) can be made to converge. In particular, $c({\varvec{q}},t)$ may be conveniently prescribed as the ratio between the classical density $D({\varvec{q}},t)$ and the Lie-transported volume element $\mu =\upeta _*\mu _0$, where $\mu _0$ is the elementary volume form in physical space. The appearance of the volume form in the denominator arises from the formal definition of Nambu–Poisson structures, as found in Vaisman (1999). Then, upon setting $\mu _0=1$ in physical space, we have

$$\begin{aligned} c=D_0\circ \varvec{\upeta } ^{-1}. \end{aligned}$$

Here, we emphasize the difference between the push-forward of the classical density $\upeta ^* D_0=(D_0/{\text {det}}\nabla \varvec{\upeta })\circ \varvec{\upeta } ^{-1}$ and the simpler composition operation $D_0\circ \varvec{\upeta } ^{-1}$, corresponding to the push-forward of the scalar function $D_0/\mu _0$. The equation of motion for $c({\varvec{q}},t)$ is thus $\partial _t c+{\varvec{u}}\cdot \nabla c=0$.

So far, nothing has been said about the evolution of the backreaction field *b* in (22). The simplest choice would involve a time-independent function. However, we will now show that this is not compatible with the structure of the original model (4)–(6). A certain insight is provided by the observation that the terms involving ${\widehat{H}}$ in (21) combine into $\int \langle \widehat{{\mathscr {D}}},{\widehat{H}}\rangle \,\textrm{d}^3q$, where

25 $$\begin{aligned} \widehat{{\mathscr {D}}}=D{\hat{\varrho }}+\frac{i\hbar }{2} {\text {div}}(c[{\hat{\varrho }},\nabla {\hat{\varrho }}\times \nabla b]) \end{aligned}$$

evidently plays the same role as the hybrid von Neumann operator $\widehat{{{\mathcal {D}}}}$ in (15). By pursuing the analogy with the original model (4)–(6), here we will insist that $\widehat{{\mathscr {D}}}$ must satisfy analogous equivariance properties to those in (16). In the case that *b* is fixed and $\widehat{{\mathscr {D}}}$ is regarded as a function of the dynamical variables $(D,{\hat{\varrho }},c)$, evidently we would have, in push-forward notation, $\widehat{{\mathscr {D}}}(\upeta _*D,\upeta _*{\hat{\varrho }},\upeta _*c)\ne \upeta _*\widehat{{\mathscr {D}}}(D,{\hat{\varrho }},c)$. Instead, if *b* is time-dependent, so that $\widehat{{\mathscr {D}}}$ depends on the four variables $(D,{\hat{\varrho }},b,c)$, then we observe that

26 $$\begin{aligned}{} & {} \widehat{{\mathscr {D}}}(\upeta _*D,\upeta _*{\hat{\varrho }}, \upeta _*b,\upeta _*c)=\upeta _*\widehat{{\mathscr {D}}}(D,{\hat{\varrho }},b,c) ,\nonumber \\{} & {} \widehat{{\mathscr {D}}}(D,{\mathscr {U}}{\hat{\varrho }} {\mathscr {U}}^\dagger ,b,c)={\mathscr {U}}\widehat{{\mathscr {D}}} (D,{\hat{\varrho }},b,c){\mathscr {U}}^\dagger . \end{aligned}$$

In particular, here we make the following prescription for the evolution of *b*:

27 $$\begin{aligned} b=b_0\circ {\varvec{\upeta }}^{-1} , \end{aligned}$$

that is *b* is also Lie transported as a scalar function. As we will see, this relation ensures conservation of the total momentum exchanged between the classical and the quantum systems.

### Quantum–Classical Fluid Equations

Having characterized the fluid Lagrangian (21) along with the evolution of the dynamical variables, we are now ready to write the equations of motion explicitly. In particular, Hamilton’s variational principle $\delta \int _{t_1}^{t_2}l\,\textrm{d}t=0$ involves the following Euler–Poincaré variations:

28 $$\begin{aligned} \delta D=-{\text {div}}(D{{\textbf{w}}}),\qquad \delta {\varvec{u}}=\partial _t{{\textbf{w}}}+{\varvec{u}}\cdot \nabla {{\textbf{w}}}-{{\textbf{w}}}\cdot \nabla {\varvec{u}}, \qquad \delta c = - {{\textbf{w}}}\cdot \nabla c, \end{aligned}$$

and

29 $$\begin{aligned} \delta { {{\hat{\varrho }}}}= & {} [{{{\hat{\upzeta }}}}, { {{\hat{\varrho }}}}] - {{\textbf{w}}}\cdot \nabla {{\hat{\varrho }}},\qquad \delta {\hat{\upxi }} = \partial _t {{{\hat{\upzeta }}}} + [ {{{\hat{\upzeta }}}}, {\hat{\upxi }} ] + {\varvec{u}}\cdot \nabla {{{\hat{\upzeta }}}} - {{\textbf{w}}}\cdot \nabla {\hat{\upxi }}, \quad \nonumber \\{} & {} \quad \delta b = - {{\textbf{w}}}\cdot \nabla b, \end{aligned}$$

where ${{\textbf{w}}}=\delta \varvec{\upeta }\circ \varvec{\upeta }^{-1}$ and ${{\hat{\upzeta }}}=\delta {\textsf{U}}\textsf{U}^{-1}\circ \varvec{\upeta }^{-1}$ are both arbitrary and vanishing at the endpoints. These variations follow directly from (23) and (27). In addition, $\delta {{\textbf{m}}}$ is arbitrary.

Before proceeding further, in this section we will make use of the partial Legendre transform ${{\textbf{m}}}=DM{\varvec{u}}$ in (21) and we will restore pressure by inserting an internal energy term of the type $-\int D{{{\mathcal {E}}}}(D)\,\textrm{d}^3 q$. While pressure effects are absent in molecular dynamics, they become important for solute–solvent coupling in solvation hydrodynamics (Bousquet et al. 2011; Hughes et al. 2012). In that context, the motion of a quantum molecule interacting with the surrounding fluid solvent cannot generally ignore the solvent pressure. Upon including the internal energy, the Lagrangian (21) becomes

30 $$\begin{aligned}{} & {} \ell (\varvec{u},D,{\hat{\upxi }},{\hat{\varrho }},b,c)\nonumber \\{} & {} \quad =\int \!\Big (\frac{M}{2}D|{\varvec{u}}|^2-D{{{\mathcal {E}}}}(D)+\big \langle {\hat{\varrho }},i\hbar D{\hat{\upxi }}-D{{\widehat{H}}}-i\hbar c\{{ {\hat{\varrho }}},{\widehat{H}}\}_b\big \rangle \Big )\textrm{d}^3q . \end{aligned}$$

In analogy with the phase-space construction in Sect. 2.1, and as a result of the relations (23) and (27), the action principle associated to this Lagrangian restricts the evolution of $(D,{{\hat{\varrho }}},b, c )$ to occur on orbits of the semidirect product ${\text {Diff}}(Q)\,\circledS \, {{{\mathcal {F}}}}(Q,{{{\mathcal {U}}}}({\textsf{H}}))$ acting on the space ${\text {Den}}(Q)\times {{{\mathcal {F}}}}(Q,{\text {Her}}({\textsf{H}}))\times {{{\mathcal {F}}}}(Q,\mathbb {R}^2)\times {{{\mathcal {F}}}}(Q,\mathbb {R}^2)$. We notice the presence of the extra variables *b* and *c* arising from the closure method and previously absent in the phase-space approach. In addition, in the present case, the Lagrangian fluid trajectories evolve along a velocity vector field ${\varvec{u}}$ that has its own equation of motion and will characterize the fluid momentum evolution. This situation differs from that in the phase-space model, where the Lagrangian paths were determined by a vector field which is given in the first equation of (12) by an algebraic expression involving the other dynamical variables. Despite these differences, the fluid system resulting from the action principle $\delta \int _{t_1}^{t_2}\ell \,\textrm{d}t=0$ has a geometric structure that is shared with common models of complex fluids (Gay-Balmaz and Ratiu 2009; Holm 2002; Tronci 2012).

While further geometric structures appearing in the present closure model will be presented later on, at the moment we are interested in the explicit form of the equations of motion. Upon denoting $\textsf{p}=D^2{{{\mathcal {E}}}}'(D)$, the action principle associated to the Lagrangian (30) leads to

31 $$\begin{aligned} \begin{aligned} MD(\partial _t+{\varvec{u}}\cdot \nabla ){\varvec{u}}&= -\nabla {\textsf{p}} -D\langle {{\hat{\varrho }}},\nabla {\widehat{H}}\rangle +\hbar \big \langle \nabla (c{{\hat{\varrho }}}), i\{{{\hat{\varrho }}},{\widehat{H}}\}_b\big \rangle +\hbar \big \langle \nabla {{\hat{\varrho }}},{i} \{c{{\hat{\varrho }}}, {\widehat{H}}\}_b\big \rangle \\&\quad -\hbar {\text {div}}\langle c{{\hat{\varrho }}},i \nabla {\hat{\varrho }}\times \nabla \widehat{H}\rangle \nabla b,\\ i\hbar (\partial _t+{\varvec{u}}\cdot \nabla ){{\hat{\varrho }}}&= \Big [{\widehat{H}}+{i\hbar }D^{-1}\Big (c\{{{\hat{\varrho }}}, {\widehat{H}}\}_b+c\{{\widehat{H}},{\hat{\varrho }}\}_b -\frac{1}{2}\big [\{c, {\widehat{H}}\}_b,{\hat{\varrho }}\big ]\Big ),{\hat{\varrho }}\Big ], \end{aligned} \end{aligned}$$

as well as

32 $$\begin{aligned} \partial _t D+{\text {div}}(D{\varvec{u}})=0,\qquad (\partial _t+{\varvec{u}}\cdot \nabla )b=0,\qquad (\partial _t+{\varvec{u}}\cdot \nabla )c=0. \end{aligned}$$

We refer to Appendix A for more calculational details. These quantum–classical fluid equations can be immediately compared to the fully quantum equations in (1). We briefly summarize our considerations as follows:

- with respect to the fully quantum treatment in (1), the quantum–classical system (31)–(32) involves two extra variables, the backreaction field *b* and the quantity *c*, which are Lie transported as scalar functions;
- the quantum potential $V_Q$ is now absent and its effects are now replaced by classical pressure effects from standard barotropic fluids;
- unlike the quantum equations in (1), the quantum–classical system (31)–(32) recovers uncoupled quantum and classical dynamics if $\nabla {\widehat{H}}=\nabla H_C\varvec{1}$, where $\varvec{1}$ is the identity matrix and $H_C$ is a scalar function;
- while the fully quantum equations in (1) contains terms of order $\hbar ^2$, only terms of the order $\hbar$ appear in the quantum–classical system (31)–(32);
- unlike the quantum equations in (1), the quantum–classical system (31)–(32) involves *only first-order gradients*;
- unlike the solvation model (3), Eqs. (31)–(32) ensure that the operator ${{\hat{\varrho }}}$ is positive semidefinite at all times. This follows from the second equation in (24);
- the system (31)–(32) may be readily extended to include a nonlocal dependence of the Hamiltonian ${\widehat{H}}$ on the variables *D* and ${{\tilde{\rho }}}=D{{\hat{\varrho }}}$, as occurring in the effective Hamiltonian ${\widehat{H}}_{\textrm{eff}}$ of the solvation model (3) in Bousquet et al. (2011).

Thus, despite their cumbersome appearance and the presence of two auxiliary frozen-in functions, the quantum–classical fluid equations represent a considerable simplification over the fully quantum hydrodynamics, where second- and third-order gradients are responsible for the appearance of interference patterns that pose severe challenges for current trajectory-based algorithms (Zhao and Makri 2003).

We also notice that, upon dropping the internal energy and setting an initially constant backreaction field, the system (31) recovers the quantum–classical Ehrenfest equations (2). In particular, the terms containing the *b*-field are unambiguously associated to the spatial inhomogeneities in the quantum state variable ${{\hat{\varrho }}}$ and these inhomogeneities are responsible for the quantum backreaction on the classical fluid flow in the current model. Furthermore, we emphasize again that the internal energy has been assumed here to depend only on the fluid density. While this is suitable for barotropic fluids, adiabatic flows that may also be realized by letting ${{{\mathcal {E}}}}={{{\mathcal {E}}}}(D,S)$ depend also on the specific entropy *S*, which is then Lie transported as an additional scalar function. On this occasion, however, we will restrict to the barotropic case for the sake of simplicity.

## Main Features and Discussion

### Mead Connection and the Stress Tensor

As we observed in Sect. 3.2, the quantum–classical von Neumann operator (25) plays the same role as its phase-space variant from Sect. 2.3. Remarkably, this operator is expressed in terms of a non-Abelian connection form. Defined on a ${\mathcal {U}}({\textsf{H}})$-bundle over the configuration manifold $Q=\mathbb {R}^3$, this quantity is usually given as $i[{{\hat{\varrho }}},\nabla {\hat{\varrho ]}}$, where the imaginary unit is inserted so that this differential one-form takes values in the space ${\text {Her}}({\textsf{H}})$ of Hermitian operators on ${\textsf{H}}$. For later convenience, here we will refer to the connection form

33 $$\begin{aligned} {\widehat{{\varvec{\Gamma }}}}=\frac{i\hbar }{2}[{{\hat{\varrho }}},\nabla {{\varrho }}] \end{aligned}$$

as the *Mead connection*. Indeed, up to a numerical factor, the same quantity first appeared in Mead’s work on geometric phases in molecular systems (Mead 1992), although its role has not been much investigated so far. Within the context of mixed quantum–classical dynamics, a phase-space extension of the Mead connection made its first appearance in the original model (4)–(6). Indeed, as shown in Gay-Balmaz and Tronci (2022a), Tronci and Gay-Balmaz (2023), Eq. (5) may be rewritten as $\varvec{{{\mathcal {X}}}}= \langle {\widehat{P}}|{{\textbf{X}}}_{\widehat{{{\mathcal {H}}}}}\rangle +\big ( \langle {{\textbf{X}}}_{{\widehat{{{\mathcal {H}}}}}}\cdot \nabla | (fJ\widehat{\varvec{\Upgamma }})\rangle -\langle f(J\widehat{\varvec{\Upgamma }})\cdot \nabla |{{\textbf{X}}}_{{\widehat{{{\mathcal {H}}}}}}\rangle \big )/f$, where $J^{jk}=\{z^j,z^k\}$, $\widehat{\varvec{\Upgamma }}={i\hbar }[{\widehat{P}},\nabla _{\!{{\textbf{z}}}}{\widehat{P}}]/2$, and we recall ${{\textbf{z}}}=({\varvec{q}},{\varvec{p}})$. As we will see in this section, the Mead connection appears again in the present fluid closure, where it is defined on the configuration space as in (33).

#### Remark 4.1

(*Mead connection in spin-orbit coupling*) The connection (33) plays a pivotal role in spin-orbit coupling (SOC), in semirelativistic quantum mechanics. In this case, the Hamiltonian operator is $\widehat{{{\mathcal {H}}}}=m^{-1}|{{\hat{{\varvec{p}}}}}|^2/2+{V({\hat{{\varvec{x}}}})}+\widehat{{{\mathcal {H}}}}_{SOC}$, where ${\hat{{\varvec{p}}}}=-i\hbar \nabla$ and

$$\begin{aligned} \widehat{{{\mathcal {H}}}}_{SOC}=-\frac{\hbar }{4 m^2 {\textsf{c}}^2}\,{\widehat{{\varvec{\sigma }}}}\times \nabla V\cdot \hat{\varvec{p}}. \end{aligned}$$

Following from the Dirac equation, the SOC operator $\widehat{ {\mathcal {H}} }_{SOC}$ represents the quantum counterpart of the Thomas precession (Baym 1969; Thomas 1927). As usual, the Pauli equation $i\hbar \partial _t\Psi =\widehat{{{\mathcal {H}}}}\Psi$ is obtained from the Dirac–Frenkel action principle $\delta \int _{t_1}^{t_2}\!\int {\text {Re}}(i\hbar \Psi ^\dagger \partial _t\Psi -\Psi ^\dagger \widehat{{{\mathcal {H}}}}\Psi )\,\textrm{d}t=0$, where $\Psi ({\varvec{x}})$ is $\mathbb {C}^2$-valued and satisfies $\int \Psi ^\dagger \Psi \,\textrm{d}^3x=1$. Following (Bialynicki-Birula et al. 1992), we exploit the exact factorization ansatz (Abedi et al. 2012) $\Psi ({\varvec{x}})=\chi ({\varvec{x}})\phi ({\varvec{x}})$. Here, $\chi \in L^2(\mathbb {R}^3)$ and $\phi ({\varvec{x}})\in \mathbb {C}^2$ satisfies $\langle \phi ({\varvec{x}}),\phi ({\varvec{x}})\rangle =1$ for all ${\varvec{x}}\in \mathbb {R}^3$. Then, the Dirac–Frenkel action principle becomes $\delta \int _{t_1}^{t_2}\!\int {\text {Re}} \big (i\hbar \chi ^*\partial _t\chi +|\chi |^2\langle \phi ,i\hbar \partial _t\phi \rangle - \langle \chi \phi ,\widehat{{{\mathcal {H}}}}(\chi \phi )\rangle \big )\textrm{d}^3 x\textrm{d}t=0$, and the SOC integrand reads

$$\begin{aligned} \langle \chi \phi ,\widehat{{{\mathcal {H}}}}_{SOC}(\chi \phi )\rangle= & {} \frac{\hbar }{4 m^2 {\textsf{c}}^2}\nabla V\times \langle {\widehat{{\varvec{\sigma }}}}\rangle \cdot {\text {Re}} (\chi ^*\hat{\varvec{p}}\chi +|\chi |^2{\textbf{A}})\nonumber \\{} & {} +\frac{\hbar }{4 m^2 {\textsf{c}}^2}|\chi |^2\langle \nabla V\times {\widehat{{\varvec{\sigma }}}},\widehat{\varvec{\Gamma }} \rangle , \end{aligned}$$

with ${\textbf{A}}:=\langle \phi ,-i\hbar \nabla \phi \rangle$. Here, we have denoted ${{\hat{\varrho }}}=\phi \phi ^\dagger$ so that the Mead connection (33) appears explicitly in the last term. The occurrence of $\widehat{\varvec{\Gamma }}$ in SOC does not seem to have appeared in the literature and a more thorough discussion is left for other venues.

As anticipated, the operator (25) can be expressed in terms of the Mead connection as $\widehat{{\mathscr {D}}} =D{{\hat{\varrho }}}+{\text {div}}(c{\widehat{{\varvec{\Gamma }}}}\times \nabla b)$, and we notice that the classical density and the quantum density matrix are given by suitable projections of $\widehat{{\mathscr {D}}}$, that is $D={\text {Tr}}\widehat{{\mathscr {D}}}$ and ${\hat{\uprho }}=\int \widehat{{\mathscr {D}}}\,\textrm{d}^3q$, respectively. Notice that, unlike other quantum–classical models widely popular in chemical physics (Bondarenko and Tempelaar 2023; Kapral 2006), here both the classical and the quantum density are positive. In more generality, the model in (31) satisfies the consistency criteria outlined at the beginning of Sect. 1.3. In particular, the third property therein involves both quantum unitary transformations and classical transformations on the configuration space (that is, cotangent lifts on phase space). The equivariance properties possessed by the Eq. (31) follow from the equivariance of their underlying variational principle, which in turn hinges on the properties (26) possessed by the von Neumann operator $\widehat{{\mathscr {D}}}$. In addition, following Proposition 5.11 in Gay-Balmaz and Tronci (2022b), the relations (26) naturally lead to

34 $$\begin{aligned} \frac{\textrm{d}{{\textbf{m}}}}{\textrm{d}t}=-\int \langle \widehat{{\mathscr {D}}},\nabla {\widehat{H}}\rangle \,\textrm{d}^3q ,\qquad i\hbar \frac{\textrm{d}{\hat{\uprho }}}{\textrm{d}t}=\int [{\widehat{H}},\widehat{{\mathscr {D}}}]\,\textrm{d}^3q. \end{aligned}$$

In the case of quantum–classical dynamics involving an infinite-dimensional quantum subsystem, we write ${\widehat{H}}({\varvec{q}})={H}({\varvec{q}},{\hat{{\varvec{x}}}},{\hat{{\varvec{p}}}})$, with $[{\hat{{\varvec{x}}}},{\hat{{\varvec{p}}}}]=i\hbar \varvec{1}$. Then, the relations above ensure momentum conservation for translation-invariant Hamiltonians such as ${\widehat{H}}({\varvec{q}})=M^{-1}{\hat{p}}^2/2+{V({\varvec{q}}-{\hat{{\varvec{x}}}})}$.

Equation (34) is verified explicitly by rewriting (31), after several rearrangements, as

35 $$\begin{aligned} \begin{aligned} MD(\partial _t+{\varvec{u}}\cdot \nabla ){\varvec{u}}&=- \langle \widehat{{\mathscr {D}}},\textrm{d}{\widehat{H}}\rangle - {\text {div}}{\textsf{T}},\\ i\hbar D(\partial _t+{\varvec{u}}\cdot \nabla ){{\hat{\varrho }}}&=[{\widehat{H}},\widehat{{\mathscr {D}}}]+{\text {div}}\! \Big (c\nabla b\times [ {\widehat{H}},{\widehat{{\varvec{\Gamma }}}}] +\frac{i\hbar }{2}c\nabla b\times \big [{\hat{\varrho }}, [{\hat{\varrho }},\nabla {\widehat{H}}]\big ]\!\Big ), \end{aligned}\nonumber \\ \end{aligned}$$

where we have introduced the stress tensor

36 $$\begin{aligned} {\textsf{T}}_{jk}= & {} \big ({\textsf{p}} -c\nabla b \cdot \big \langle {\widehat{{\varvec{\Gamma }}}}, \times \nabla {\widehat{H}}\big \rangle \big )\delta _{jk}+c\langle {\widehat{{\varvec{\Gamma }}}},\times \nabla {\widehat{H}}\rangle _j\partial _k b+c\langle ( \nabla b\times {\widehat{{\varvec{\Gamma }}}})_j, \partial _k{\widehat{H}}\rangle \nonumber \\{} & {} + c\langle (\nabla {\widehat{H}}\times \nabla b)_j,{\widehat{\Gamma }}_k\rangle . \end{aligned}$$

One verifies that ${\textsf{T}}$ is symmetric by using the argument that any matrix of the form $({\textbf{a}}\times {\textbf{b}}){\textbf{c}}^T+({\textbf{c}}\times {\textbf{a}}){\textbf{b}}^T+({\textbf{b}}\times {\textbf{c}}){\textbf{a}}^T$ is symmetric by the Jacobi identity. As shown in Appendix B, the symmetry of ${\textsf{T}}$ follows from the fact that $\langle {{\hat{\varrho }}},i\hbar \{ {\hat{\varrho }}, {\widehat{H}}\}_b\rangle$ is invariant under rotations of $\nabla {\widehat{H}}$, $\nabla {\hat{\varrho }}$, and $\nabla b$. Notice that this property would fail if the evolution Eq. (27) was dropped and the backreaction field *b* was treated as a constant: in that case, the last relation in (29) would be replaced by $\delta b=0$, so that the resulting stress tensor would fail to be symmetric. In addition, we observe that the Mead connection plays a predominant role not only in the construction of the hybrid von Neumann operator, but also in the characterization of the fluid stresses. Before concluding this section, we observe that the momentum equation can be further rearranged as

$$\begin{aligned} MD(\partial _t+{\varvec{u}}\cdot \nabla ){\varvec{u}}= & {} -D \langle {{{\hat{\varrho }}}},\textrm{d}{\widehat{H}}\rangle - \langle (c{\widehat{{\varvec{\Gamma }}}}\times \nabla b)\cdot \nabla ,\nabla {\widehat{H}}\rangle +\langle (\nabla {\widehat{H}}\times \nabla b)\cdot \nabla ,c{\widehat{{\varvec{\Gamma }}}}\rangle \\{} & {} - \nabla ({\textsf{p}}-c\langle {{\hat{\varrho }}},\{{{\hat{\varrho }}},{\widehat{H}}\}_b\rangle )-{\text {div}}(c\langle {\widehat{{\varvec{\Gamma }}}},\times \nabla {\widehat{H}}\rangle (\nabla b)^T), \end{aligned}$$

and, using the definition (33), we notice certain similarities between the first line above and the expression of the original phase-space vector field (5).

### Hamiltonian Structure and Cross-Helicity

Having characterized the quantum–classical fluid system, its stress tensor, and the geometric quantities appearing therein, in this section we will show how more insight can be obtained by looking at the underlying Hamiltonian structure. As usual, the latter is given in terms of a Poisson structure and a Hamiltonian functional which identifies the energy of the system.

The Hamiltonian structure of the fluid system (31) may be easily found by considering the action principle $\delta \int _{t_1}^{t_2}l\,\textrm{d}t=0$ associated to (21) and introducing the convenient variable ${{\tilde{\rho }}}=D{{\hat{\varrho }}}$. Then, we have $\delta \int _{t_1}^{t_2}\!\big (\int ({{\textbf{m}}}\cdot {\varvec{u}}+\langle {{\tilde{\rho }}},i\hbar {\hat{\upxi }}\rangle )\,\textrm{d}^3 q-h\big )\textrm{d}t=0$, where the Hamiltonian functional reads

37 $$\begin{aligned} h({{\textbf{m}}},{{\tilde{\rho }}},D,b,c)=\int \!\bigg (\frac{|{{\textbf{m}}}|^2}{2MD}+D{{{\mathcal {E}}}}(D)+\bigg \langle {{\tilde{\rho }}},{{\widehat{H}}}+\frac{i\hbar }{D^2} c\{{ {{\tilde{\rho }}}},{\widehat{H}}\}_b\bigg \rangle \bigg )\,\textrm{d}^3q. \end{aligned}$$

Notice that here we have restored the internal energy in (21) in order to retain pressure effects. The candidate Poisson structure is found by applying the relation ${\dot{f}}=\{f,h\}$ and using the equations of motion associated to an arbitrary Hamiltonian. This step leads to the bracket

38 $$\begin{aligned} \{f,k\}({{\textbf{m}}},{{\tilde{\rho }}},D,b,c)&=\int \!{{\textbf{m}}}\cdot \left( \frac{\delta k}{\delta {{\textbf{m}}}}\cdot \nabla \frac{\delta f}{\delta {{\textbf{m}}}}-\frac{\delta f}{\delta {{\textbf{m}}}}\cdot \nabla \frac{\delta k}{\delta {{\textbf{m}}}}\right) \textrm{d}^3 q\nonumber \\&\quad - \int \! c{\text {div}}\!\left( \frac{\delta f}{\delta {{\textbf{m}}}}\frac{\delta k}{\delta c}-\frac{\delta k}{\delta {{\textbf{m}}}}\frac{\delta f}{\delta c}\right) \textrm{d}^3 q\nonumber \\&\quad - \int \!D\left( \frac{\delta f}{\delta {{\textbf{m}}}}\cdot \nabla \frac{\delta k}{\delta D}-\frac{\delta k}{\delta {{\textbf{m}}}}\cdot \nabla \frac{\delta f}{\delta D}\right) \textrm{d}^3 q \nonumber \\&\quad - \int \! b{\text {div}}\!\left( \frac{\delta f}{\delta {{\textbf{m}}}}\frac{\delta k}{\delta b}-\frac{\delta k}{\delta {{\textbf{m}}}}\frac{\delta f}{\delta b}\right) \textrm{d}^3 q \nonumber \\&\quad -\int \left\langle {{\tilde{\rho }}},i\hbar ^{-1}\left[ \frac{\delta k}{\delta {{\tilde{\rho }}}},\frac{\delta h}{\delta {{\tilde{\rho }}}}\right] +\frac{\delta f}{\delta {{\textbf{m}}}}\cdot \nabla \frac{\delta k}{\delta {{\tilde{\rho }}}}-\frac{\delta k}{\delta {{\textbf{m}}}}\cdot \nabla \frac{\delta f}{\delta {{\tilde{\rho }}}}\right\rangle \textrm{d}^3 q, \end{aligned}$$

which is Lie–Poisson on the dual of the semidirect-product Lie algebra $\big ({\mathfrak {X}}(\mathbb {R}^3)\,\circledS \, {{{\mathcal {F}}}}(\mathbb {R}^3,{\mathfrak {u}}(\textsf{H}))\big )\,\circledS \,\big ({{{\mathcal {F}}}}(\mathbb {R}^3) \times {{{\mathcal {F}}}}(\mathbb {R}^3)\times {\text {Den}}(\mathbb {R}^3)\big )$. Here, ${\mathfrak {X}}(\mathbb {R}^3)$ denotes the space of vector fields on $\mathbb {R}^3$, while ${\mathfrak {u}}({\textsf{H}})$ identifies the Lie algebra of skew-Hermitian operators on the quantum Hilbert space ${\textsf{H}}$. We observe that this bracket structure is similar to that underlying the fully quantum hydrodynamic Eq. (1), although in that case the second and fourth lines are absent. Lie–Poisson structures of the type (38) have appeared in several instances over the years, in the context of complex fluid models (Gay-Balmaz and Ratiu 2009).

The bracket (38) possesses the Casimir invariants

39 $$\begin{aligned} C_1={\text {Tr}}\int \! D F(D^{-1}{{\tilde{\rho }}})\textrm{d}^3q \qquad \text { and }\qquad C_2=\int \!D\Phi (b,c,\{\Lambda _n\})\,\textrm{d}^3q, \end{aligned}$$

where

$$\begin{aligned} \Lambda _n=(D^{-1}\nabla c\times \nabla b\cdot \nabla )^n\Vert D^{-1}{{\tilde{\rho }}}\Vert ,\qquad \ n=1,2,\ldots \end{aligned}$$

satisfies the transport equation $\partial _t\Lambda _n+{\varvec{u}}\cdot \nabla \Lambda _n=0$ (Henyey 1982), while $\Phi$ and *F* are an arbitrary scalar function and an arbitrary matrix analytic function, respectively. We remark that the Casimirs $C_2$ extend a similar class of invariants appearing in the dynamics of ferromagnetic fluids (Holm and Kupershmidt 1988). While here we treat $C_1$ and $C_2$ separately for convenience, we notice that they may be combined into only one functional of the type ${\text {Tr}}\int \! D \textsf{F}(b,c,\Lambda _n,D^{-1}{{\tilde{\rho }}})\textrm{d}^3q$.

We will now show that more insight may be obtained upon writing ${{\tilde{\rho }}}=D\psi \psi ^\dagger$, in which case $C_1$ becomes a trivial constant and $\Lambda _n$ vanishes, so that $C_2=\int \!D\Phi (b,c)\,\textrm{d}^3q$. From the first two in (23), we find that the evolution of $\psi (t)$ is $\psi =({\textsf{U}}\psi _0) \circ \varvec{\upeta }^{-1}$, so that the time derivative gives $\partial _t\psi ={\hat{\upxi \psi }}-{\varvec{u}}\cdot \nabla \psi$, and thus

$$\begin{aligned} \langle {{\tilde{\rho }}},i\hbar {\hat{\upxi \rangle }} =D\langle \psi ,i\hbar \partial _t\psi \rangle -D{\varvec{u}}\cdot {\textbf{A}},\qquad \ \text { with }\qquad \, {\textbf{A}}=\langle \psi ,-i\hbar \nabla \psi \rangle \end{aligned}$$

being the *Berry connection* (Berry 1984). With this substitution and the introduction of the canonical fluid momentum ${{\textbf{M}}}={{\textbf{m}}}-D{\textbf{A}}$, the phase-space Lagrangian (21) becomes $\int ({{\textbf{M}}}\cdot {\varvec{u}}+D\langle \psi ,i\hbar \partial _t\psi \rangle )\,\textrm{d}^3 q-{h}$. Also, upon retaining the internal energy term, the Hamiltonian functional reads

40 $$\begin{aligned} h({{\textbf{M}}},\psi ,D,b,c)= & {} \int \Bigg (\frac{|{{\textbf{M}}}+D{\textbf{A}}|^2}{2MD}+D{{{\mathcal {E}}}}(D)\nonumber \\ {}{} & {} +\big \langle \psi ,D{{\widehat{H}}}\psi +c\nabla b\cdot \nabla {\widehat{H}}\times (i\hbar \nabla +{\textbf{A}})\psi \big \rangle \Bigg )\,\textrm{d}^3q. \end{aligned}$$

In this case, since $\delta \psi$ and $\delta {{\textbf{M}}}$ are arbitrary, and ${{\textbf{M}}}=D(M{\varvec{u}}-{\textbf{A}})$, Eq. (31) become

41 $$\begin{aligned} MD(\partial _t+{\varvec{u}}\cdot \nabla ){\varvec{u}}&= -{{\textbf{E}}}-{\varvec{u}}\times {{\textbf{B}}}-\nabla {{\textsf{p}}} -D\nabla \langle {\widehat{H}}\rangle + \langle \psi ,i\hbar \nabla b\cdot \widetilde{{\textbf{F}}} \times \nabla \psi \rangle \nabla c\\&\quad +{\text {div}}\langle \psi ,i\hbar c \widetilde{{\textbf{F}}}\times \nabla \psi \rangle \,\nabla b \nonumber \end{aligned}$$

42 $$\begin{aligned} i\hbar D(\partial _t+{\varvec{u}}\cdot \nabla )\psi&= D{\widehat{H}}\psi +{i\hbar }c\bigg (\{\psi \psi ^\dagger , {\widehat{H}}\}_b+\{{\widehat{H}},\psi \psi ^\dagger \}_b -\frac{1}{2c}\big [\{c, {\widehat{H}}\}_b,\psi \psi ^\dagger \big ]\bigg )\psi , \end{aligned}$$

where we have denoted

$$\begin{aligned} {{\textbf{E}}}=-\partial _t{\textbf{A}}-\nabla \langle \psi , i\hbar \partial _t\psi \rangle ,\qquad \quad {{\textbf{B}}}=\nabla \times {\textbf{A}},\qquad \quad \widetilde{{\textbf{F}}}=-\big ({\nabla {\widehat{H}}}-\langle \psi ,{\nabla {\widehat{H}}}\psi \rangle \big ). \end{aligned}$$

Notice that Eq. (32) remains unchanged. Besides the usual Lorentz force, well known in molecular dynamics (Agostini et al. 2016), we observe the presence of an extra hydrodynamic force involving the *fluctuation force operator* $\widetilde{{\textbf{F}}}$ which identifies the quantum fluctuations around the Hellman–Feynman average $\langle \widehat{{\textbf{F}}}\rangle =-\langle \psi ,{\nabla {\widehat{H}}}\psi \rangle$ (Feynman 1939). We remark that the last term in the momentum equation above is exactly the same as the last term in the first equation of (31), although the use of the conditional state vector $\psi$ now unfolds the occurrence of the fluctuation force. This occurrence in the present fluid model does not come as a surprise, since the same quantity already appears in the phase-space model (4)–(6), as shown in (Gay-Balmaz and Tronci 2022a).

Importantly, the momentum equation above can be written in terms of the Lie-derivative as $(\partial _t+\pounds _{\varvec{u}})(M{\varvec{u}}-{\textbf{A}})=-\nabla (\delta h/\delta D)+ D^{-1}(\delta h/\delta c)\nabla c+D^{-1}(\delta h/\delta b)\nabla b$. On the one hand, upon computing $\delta h/\delta b= {\text {div}}\langle \psi ,i\hbar c\widetilde{{\textbf{F}}}\times \nabla \psi \rangle$ and $\delta h/\delta c=\nabla b\cdot \langle \psi ,i\hbar \widetilde{{\textbf{F}}}\times \nabla \psi \rangle$ this leads to the following circulation dynamics:

$$\begin{aligned} \frac{\textrm{d}}{\textrm{d}t}\oint _{\varvec{\gamma }}(M{\varvec{u}}-{\textbf{A}})\cdot \textrm{d}{\varvec{q}}{} & {} =\oint _{\varvec{\gamma }}\frac{1}{D}\Big ({\text {div}}\langle \psi ,i\hbar c\widetilde{{\textbf{F}}}\times \nabla \psi \rangle \,\nabla b\\ {}{} & {} \quad +\langle \psi ,i\hbar \nabla b\cdot \widetilde{{\textbf{F}}}\times \nabla \psi \rangle \nabla c\Big ) \cdot \textrm{d}{\varvec{q}}, \end{aligned}$$

where $\varvec{\gamma }=\varvec{\upeta } \circ \varvec{\gamma }_0$ is any loop moving with the Lagrangian fluid path $\varvec{\upeta }$. The Lie-derivative form of the momentum equation reads equivalently as $D(\partial _t+\pounds _{\varvec{u}})(M{\varvec{u}}-{\textbf{A}})=-D\nabla (\delta h/\delta D) + (\delta h/\delta b)\nabla b+(\delta h/\delta c)\nabla c$. Consequently, taking the dot product with $\nabla c\times \nabla b$, one obtains the *cross-helicity* invariant

43 $$\begin{aligned} C_3=\int (M{\varvec{u}}-{\textbf{A}})\cdot \nabla c\times \nabla b\,\textrm{d}^3q. \end{aligned}$$

Here, the name follows from similar invariants appearing in the hydrodynamics of magnetized plasmas (Calkin 1963). An even more similar expression arises in hybrid kinetic-fluid models (Tronci et al. 2015). Notice that, in the case of the Ehrenfest fluid model (2), the entire hydrodynamic helicity $\int (M\varvec{u}-\textbf{A})\cdot \nabla \times (M\varvec{u}-\textbf{A}) \textrm{d}^3q$ is conserved.

We have seen how the use of the conditional state vector $\psi$, while unfolding the role of the fluctuation force $\widetilde{\textbf{F}}$, also allows for a systematic characterization of circulation and cross-helicity. All these Casimirs may be used, for example, for a Lyapunov stability study via the energy-Casimir method (Holm et al. 1985).

### Pure-Dephasing Systems

As an informative specialization of our fluid model (31)–(32), in this section we consider a particular type of quantum–classical systems, recently studied in Manfredi et al. (2023). In the fully quantum formulation, a *pure-dephasing* system is given by a Hamiltonian operator of the type $\widehat{{{\mathcal {H}}}}={H}_0({\hat{{\varvec{x}}}},{\hat{{\varvec{p}}}})+{H}_I({\hat{{\varvec{x}}}},{\hat{{\varvec{p}}}},{\widehat{A}})$, where ${\widehat{A}}$ is an operator commuting with the canonical observables $({\hat{{\varvec{x}}}},{\hat{{\varvec{p}}}})$. For the present discussion, it is convenient to make extensive use of the notation $\langle {\widehat{A}}\rangle =\langle {{\hat{\varrho }}},{\widehat{A}}\rangle$. Also, for the sake of simplicity, here we will consider the case when ${\widehat{A}}$ is a given Pauli matrix ${\widehat{\sigma }}_k$ and the fully quantum Hamiltonian is given as

44 $$\begin{aligned} \widehat{{{\mathcal {H}}}}=\frac{1}{2M}|{{\hat{{\varvec{p}}}}}|^2 +V_0({{\hat{{\varvec{x}}}}}) +{V}_I({\hat{{\varvec{x}}}}){\widehat{\sigma }}_k, \end{aligned}$$

We will now present a comparison of results obtained from the study of pure-dephasing dynamics in quantum hydrodynamics, the Ehrenfest fluid model, and quantum–classical hydrodynamics. As we will see, the latter succeeds in retaining the quantum backreaction on the classical flow, which instead is lost in Ehrenfest dynamics.

In first place, we specialize the quantum hydrodynamics equations to the Hamiltonian (44), so that upon replacing ${\widehat{H}}=V_0\varvec{1} +{V}_I{\widehat{\sigma }}_k$ in (1), we have

45 $$\begin{aligned} \begin{aligned} M(\partial _t + {\varvec{u}}\cdot \nabla ){\varvec{u}}&= - \nabla (V_Q+V_0)-\langle {\widehat{\sigma }}_k\rangle \nabla V_I -\frac{\hbar ^2}{2MD}\partial _j\langle D \nabla {\hat{\varrho }}, \partial _j{\hat{\varrho }}\rangle ,\\ i\hbar D(\partial _t +{\varvec{u}}\cdot \nabla ){\hat{\varrho }}&= DV_I[{\widehat{\sigma }}_k,{\hat{\varrho }}]+\frac{\hbar ^2}{2M}\textrm{div}(D[{\hat{\varrho }},\nabla {\hat{\varrho }}]) ,\qquad \partial _t D+\textrm{div}(D{\varvec{u}})=0\,, \end{aligned} \end{aligned}$$

where we have rearranged the quantum evolution equation. We observe that the overall expectation value $\langle \!\langle {\widehat{\sigma }}_k\rangle \!\rangle =\int \!D\langle {\widehat{\sigma }}_k\rangle \,\textrm{d}^3 r$ remains constant in time, thereby ensuring physical consistency. In particular, the initial condition $\langle \!\langle {\widehat{\sigma }}_k\rangle \!\rangle =0$ is preserved by the dynamics. Nevertheless, the same does not hold for the local expectation $\langle {\widehat{\sigma }}_k\rangle$, which indeed has nontrivial dynamics. This means that the quantum degrees of freedom feed back in the hydrodynamic flow via both last two force terms in the momentum equation. Thus, despite the simplicity of our pure-dephasing Hamiltonian (44), the full quantum hydrodynamics equations remain rather challenging.

The situation changes drastically in the case of the Ehrenfest fluid model. Indeed, if we replace ${\widehat{H}}=V_0\varvec{1} +{V}_I{\widehat{\sigma }}_k$ in (2), we have

$$\begin{aligned}{} & {} M(\partial _t + {\varvec{u}}\cdot \nabla ){\varvec{u}}= - \nabla V_0 -\langle {\widehat{\sigma }}_k\rangle \nabla V_I,\qquad \ i\hbar (\partial _t +{\varvec{u}}\cdot \nabla ){\hat{\varrho }} = V_I[{\widehat{\sigma }}_k,{\hat{\varrho }}],\\{} & {} \partial _t D+\textrm{div}(D{\varvec{u}})=0. \end{aligned}$$

In this case, it is clear that the initial condition $\langle {\widehat{\sigma }}_k\rangle =0$ is preserved by the dynamics. This means that the hydrodynamic flow decouples entirely from the quantum motion, so that the Ehrenfest model fails to capture any quantum backreaction in pure-dephasing systems (Manfredi et al. 2023).

Finally, let us now consider the quantum–classical fluid model in (31)–(32). If we replace ${\widehat{H}}=V_0\varvec{1} +{V}_I{\widehat{\sigma }}_k$ in (32), we obtain

$$\begin{aligned} MD(\partial _t+{\varvec{u}}\cdot \nabla ){\varvec{u}}&=- \nabla {\textsf{p}}- D\nabla V_0 -D\langle {\widehat{\sigma }}_k\rangle \nabla V_I +\frac{\hbar }{2} \big \langle \nabla (c{{\hat{\varrho }}}), i\{[{{\hat{\varrho }}},{\widehat{\sigma }}_k],V_I\}_b\big \rangle \\&\quad +\frac{\hbar }{2} \big \langle \nabla {{\hat{\varrho }}},{i} \{c[{{\hat{\varrho }}},{\widehat{\sigma }}_k],V_I\}_b\big \rangle -\frac{\hbar }{2} {\text {div}}\langle {{\hat{\varrho }}},ic \nabla [{{\hat{\varrho }}},{\widehat{\sigma }}_k]\times \nabla V_I\rangle \nabla b\\ i\hbar D(\partial _t+{\varvec{u}}\cdot \nabla ){{\hat{\varrho }}}&= DV_I\big [{\widehat{\sigma }}_k,{{\varrho }\big ]}+{i\hbar }c \big [\{V_I,[{\widehat{\sigma }}_k,{{\varrho }}]\}_b,{{\varrho }}\big ]-\frac{i\hbar }{2}\{c, V_I\}_b\big [\big [{\widehat{\sigma }}_k,{{\varrho }}\big ],{{\varrho }\big ]}. \end{aligned}$$

Upon noticing that $\big \langle {\widehat{\sigma }}_k,\big [\{V_I,[{\widehat{\sigma }}_k,{\hat{\varrho }}]\}_b,{{\varrho }\big ]\big \rangle } =\{V_I,\Vert [{\widehat{\sigma }}_k,{{\varrho }}]\Vert ^2\}_b/2$, we see that, in the case ${{\hat{\varrho }}}=\psi \psi ^\dagger$, standard properties of the Pauli matrices lead to $\Vert [{\widehat{\sigma }}_k,{{\varrho }}]\Vert ^2=2(1-\langle {\widehat{\sigma }}_k\rangle ^2)$ and thus $D(\partial _t+{\varvec{u}}\cdot \nabla )\langle {\widehat{\sigma }}_k\rangle =\{V_I, c(1-\langle {\widehat{\sigma }}_k\rangle ^2)\}_b$. This dynamics is generally nontrivial, so that, unlike the Ehrenfest model and similarly to the quantum case, the initial value $\langle {\widehat{\sigma }}_k\rangle$ is not generally preserved in time. This means that the quantum evolution keeps feeding back into the classical flow. However, unlike quantum hydrodynamics, the fluid flow decouples entirely in the case when $V_I$ is spatially constant, in agreement with the item (4) of the consistency criteria discussed in the second paragraph of Sect. 1.3.

The present study has shown that the quantum–classical hydrodynamic model (31)–(32) overcomes the problematic force cancelations inherited from Ehrenfest dynamics. Indeed, in the new model the quantum backreaction persists through the force terms containing the backreaction field *b*, which then acquires a fundamental role. While the persistence of backreaction is shared with quantum hydrodynamics, the absence of second- and third-order gradients in the model Eqs. (31)–(32) represents a substantial difference, which may lead to important simplifications from the viewpoint of both numerical and functional analysis.

Before concluding this section, we emphasize that, while emerging as drastic simplifications of more realistic problems, pure-dephasing systems have been considered in a variety of different fields, from optical physics (Blais et al. 2004), to quantum chemistry (Reichman et al. 1996), and solvation dynamics (Hughes et al. 2012). In the context of solvation hydrodynamics, quantum–classical pure-dephasing Hamiltonians were considered in Hughes et al. (2012), although in that case the purely classical potential $V_0$ is replaced by a nonlocal convolution of the solvent density *D*. While this takes the problem to a higher level of difficulty, none of the previous arguments would change in the presence of nonlocal potentials.

### Invariant Planar Subsystems

In the search for a simplified version of the quantum–classical fluid Eqs. (31)–(32), it is instructive to look for a lower-dimensional invariant subsystem. While we observe that the quantum backreaction is lost if we specialize our model to one spatial dimension, the same is not true for the two-dimensional case. Indeed, if we restrict to consider a planar flow with ${\varvec{u}}=({\varvec{v}},0)$ and ${\varvec{v}}\in {\mathfrak {X}}(\mathbb {R}^2)$, then $b(x,y,z,t)=\beta z$ identifies an exact solution of the second equation in (32). If all the other variables are restricted to depend only on the planar coordinates, then the Eqs. (31)–(32) become

46 $$\begin{aligned} MD(\partial _t+{\varvec{v}}\cdot \nabla ){\varvec{v}}&= -\nabla {\textsf{p}} -\langle {{\hat{\varrho }}},D\nabla {\widehat{H}}\rangle +\hbar \big \langle \nabla ({\tilde{c}}{{\hat{\varrho }}}),i\{{{\hat{\varrho }}}, {\widehat{H}}\}\big \rangle +\hbar \big \langle \nabla {{\hat{\varrho }}},{i} \{{\tilde{c}} {{\hat{\varrho }}}, {\widehat{H}}\}\big \rangle ,\nonumber \\ i\hbar D(\partial _t+{\varvec{v}}\cdot \nabla ){{\hat{\varrho }}}&= \Big [D{\widehat{H}}+{i\hbar }\Big ({\tilde{c}}\{{{\hat{\varrho }}}, {\widehat{H}}\}+{\tilde{c}}\{{\widehat{H}},{\hat{\varrho }}\}-\frac{1}{2}\big [\{{\tilde{c}}, {\widehat{H}}\},{\hat{\varrho }}\big ]\Big ),{\hat{\varrho \Big ]}},\nonumber \\ \partial _t D+\textrm{div}(D{\varvec{v}})&= 0 \,,\qquad \quad \partial _t {\tilde{c}}+{\varvec{v}}\cdot \nabla {\tilde{c}}=0\,, \end{aligned}$$

where we have denoted ${\tilde{c}}=\beta c$ and $\{A,B\}={\textbf{e}}_3\cdot \nabla A\times \nabla B=\partial _xA\partial _y B-\partial _yA\partial _x B$. Here, no confusion should arise with the canonical phase-space bracket used in earlier sections. As it stands, the only substantial difference between the above planar subsystem and the full 3D model (31)–(32) is that the backreaction field has now become merely a numerical parameter $\beta$, now incorporated in the field ${\tilde{c}}=\beta c$. Nevertheless, the backreaction terms persist in the momentum equation, with the exception of the vertical forces.

We notice that these equations are again Hamiltonian with the Poisson bracket given by (38) without the terms in the fourth line, and the Hamiltonian (37) with the replacement $c\{,\,\}_b\rightarrow {\tilde{c}}\{,\,\}$ in the last term. This means that the functional $C_1$ in (39) is still a dynamical invariant, while the second Casimir drops to $C_2=\int D\Phi ({\tilde{c}},\{\Lambda _n\})\,\textrm{d}^3q$. In turn, upon considering the case ${{\hat{\varrho }}}=\psi \psi ^\dagger$, the third invariant (43) becomes $C_3=\int \!\Omega \Theta ({\tilde{c}})\,\textrm{d}^3q$, where $\Omega ={\textbf{e}}_3\cdot \nabla \times (M{\varvec{v}}-{\textbf{A}})=\omega +\hbar {\text {Im}}\{\psi ^\dagger ,\psi \}$ is the canonical vorticity and $\Theta$ is an arbitrary function. In particular, we have $\nabla \times {\textbf{A}}=B{\textbf{e}}_3$ with $B=\hbar {\text {Im}}\{\psi ^\dagger ,\psi \}$, and the equations of motion (41) resulting in the case ${{\hat{\varrho }}}=\psi \psi ^\dagger$ reduce to

47 $$\begin{aligned} M(\partial _t+{\varvec{v}}\cdot \nabla ){\varvec{v}}&= -{{\textbf{E}}}- B{\varvec{v}}\times {\textbf{e}}_3 -D^{-1}\nabla {\textsf{p}}-\nabla \langle {\widehat{H}}\rangle +D^{-1} \langle \psi ,i\hbar \textbf{e}_3\cdot \widetilde{{\textbf{F}}} \times \nabla \psi \rangle \nabla {\tilde{c}},\nonumber \\ i\hbar (\partial _t+{\varvec{v}}\cdot \nabla )\psi&= {\widehat{H}}\psi +{i\hbar }\frac{{\tilde{c}}}{D} \Big (\{\psi \psi ^\dagger ,{\widehat{H}}\}+\{{\widehat{H}}, \psi \psi ^\dagger \}-\frac{1}{2}\big [\{\ln {\tilde{c}}, {\widehat{H}}\},\psi \psi ^\dagger \big ]\Big )\psi , \nonumber \\ \partial _tD+{\text {div}}(D{\varvec{v}})&=0\,,\qquad \qquad \partial _t{\tilde{c}}+{\varvec{v}}\cdot \nabla {\tilde{c}}=0. \end{aligned}$$

In this case, we have the following circulation law:

$$\begin{aligned} \frac{\textrm{d}}{\textrm{d}t}\oint _{\varvec{\gamma }}(M{\varvec{v}}-{\textbf{A}})\cdot \textrm{d}{\varvec{q}}= \oint _{\varvec{\gamma }}\frac{1}{D}\langle \psi ,i\hbar \textbf{e }_3\cdot \widetilde{{\textbf{F}}}\times \nabla \psi \rangle \nabla {\tilde{c}}\cdot \textrm{d}{\varvec{q}}, \end{aligned}$$

where $\varvec{\gamma }(t)$ is again an arbitrary planar loop moving with the Lagrangian fluid flow.

In the attempt to further simplify our quantum–classical fluid equations, we now consider the case of an incompressible fluid flow. In this case, we have ${\text {div}}{\varvec{v}}=0$ and the volume form $\textrm{d}^3q$ in physical space is preserved in time. For example, this situation could apply to an incompressible fluid solvent interacting with a quantum solute molecule. Then, since ${\tilde{c}}=\beta D$ (recall the discussion in Sect. 3.2), Eq. (47) become

48 $$\begin{aligned} M(\partial _t+{\varvec{v}}\cdot \nabla ){\varvec{v}}&= -{{\textbf{E}}}- B{\varvec{v}}\times {\textbf{e}}_3 -\nabla {\textsf{p}} + \beta \langle \psi ,i\hbar \textbf{e }_3\cdot \widetilde{{\textbf{F}}}\times \nabla \psi \rangle \nabla \ln D,\nonumber \\ i\hbar (\partial _t+{\varvec{v}}\cdot \nabla )\psi&= {\widehat{H}}\psi +{i\hbar }\beta \Big (\{\psi \psi ^\dagger , {\widehat{H}}\}+\{{\widehat{H}},\psi \psi ^\dagger \}-\frac{1}{2}\big [\{\ln D, {\widehat{H}}\},\psi \psi ^\dagger \big ]\Big )\psi , \nonumber \\ \partial _tD+{\varvec{v}}\cdot \nabla D&=0. \end{aligned}$$

Here, the pressure $\mathsf p$ is now a Lagrange multiplier enforcing the condition ${\text {div}}{\varvec{v}}=0$. We recognize that, even in the incompressible two-dimensional case, the backreaction forces persist and this is true also for pure-dephasing systems. Similarly to the case treated in the previous paragraph, the system (48) possesses the Casimir invariant ${{{\mathcal {C}}}}=\int (\Omega \Theta (D)+\Phi (D))\,\textrm{d}^3q$.

## Conclusions and Perspectives

The idea of restoring trajectories in quantum dynamics is extremely inviting, as it offers the possibility of new convenient computational schemes borrowing methods from classical simulation codes. However, despite the considerable work carried out over the last few decades on Madelung’s hydrodynamics, the latter possesses severe difficulties that continue to challenge the community. In this scenario, mixed quantum–classical models represent a promising perspective. While most of these models continue to be used in several test cases with a certain success, their underlying equations suffer from well-known consistency issues. The possible violation of Heisenberg’s uncertainty principle in the most popular method (Bondarenko and Tempelaar 2023) is only one example (Agostini et al. 2007).

Having proposed a new Hamiltonian phase-space model overcoming these issues, here we have dealt with the problem of formulating a Hamiltonian fluid closure which goes beyond Ehrenfest dynamics and yet satisfies its consistency properties. As we showed, the proposed complex fluid model succeeds in capturing quantum–classical correlations in the case of pure-dephasing systems, which is precisely where the Ehrenfest model fails. Certain pure-dephasing systems were shown to be challenging also for other models alternative to Ehrenfest (Bondar et al. 2019). Importantly, the proposed model has also the advantage of involving only first-order gradients. This point represents a major simplification over Madelung hydrodynamics, whose main difficulties arise from the appearance of higher-order gradients in its equations of motion. This simplification happens at the expense of introducing two extra scalar fields that are transported by the flow. While the field *c* is naturally linked to the fluid density, the backreaction field *b* deserves further attention. Indeed, it is not clear how *b* should be initialized. One has a few possibilities in this regard.

A possible way to initialize *b* consists in adopting a further closure, that is expressing it as a transported scalar that is constructed from the remaining variables. For example, the quantity ${\text {Tr}} F({{\hat{\varrho }}})$ is transported for any matrix analytic function *F* and one may set $b={\text {Tr}} F({{\hat{\varrho }}})$ for some *F*. For example, one may think of setting $b=\alpha {\text {Tr}} ({{\hat{\varrho }\ln {{\hat{\varrho }}}}})$, for some parameter $\alpha$. The use of entropy functionals in hydrodynamic closures of mixed quantum–classical dynamics was first proposed in Bousquet et al. (2011). More simply, one may also set $b=\Vert {\hat{\varrho }}\Vert ^2$. However, if ${{\hat{\varrho }}}$ is initialized as a projection, these expressions reduce to a constant thereby eliminating the backreaction. A possible alternative is to initialize *b* by its equilibrium value $b_e$. Simple equilibria of the model Eqs. (31)–(32) may be easily found by setting $\delta (h+C)=0$, where *C* is one of the Casimirs treated in Sect. 4.2, or any combination thereof. For example, take the second in (39) with the particular choice $C=\int D\Phi (b)\,\textrm{d}^3 q$, where $\Phi$ is an arbitrary function. Since we are interested in the equilibria of *b*, we can simply set $\delta (h+C)/\delta b=0$. Absorbing the constants into $\Phi$, we obtain $\Phi '(b_e)={\text {div}}\langle c_e{{\varrho }_e},i \nabla {\hat{\varrho }}_e\times \nabla \widehat{H}\rangle /D_e$. Then, assuming that $\Phi '$ is invertible, we find that $b_e$ must be a function of the quantity ${\text {div}}\langle c_e{{\varrho }_e},i \nabla {\hat{\varrho }}_e\times \nabla \widehat{H}\rangle /D_e$, thereby characterizing a possible initial profile of the backreaction field.

As we have seen, interesting connections to spin-orbit coupling emerge and this is a completely unexplored direction, suggesting that quantum–classical coupling may be modeled by the same algebraic structures appearing in the semirelativistic limit of the Dirac equation. Then, a natural open question concerns the comparison between the fully quantum treatment of spin-orbit coupling in quantum hydrodynamics and the analogue treatment in mixed quantum–classical dynamics. Addressing this question requires extending the present model to the case in which the coupling depends on the momentum. This extension is currently under development.

Finally, one may wonder about possible numerical implementations of the proposed model. Given the simpler level of difficulty, we plan to start our computational efforts by focusing on the planar subystems. As the backreaction field is absorbed into a constant number, not only does this case allow for less computational resources, but also eliminates the necessity of dealing with the question about the initial profile of *b*. Given the presence of several advection equations and a variational formulation, this case would also offer a testing ground for recent structure-preserving finite-element schemes developed within our groups for the long-time simulation of fluid problems (Gawlik and Gay-Balmaz 2021).

## Calculational Details on the Model Equations

We give here some details on the derivation of the quantum–classical fluid Eq. (31) from the variational principle $\delta \int _{t_1}^{t_2}\ell \textrm{d} t=0$ with variations (28)–(29). First, we note that for an arbitrary Lagrangian $\ell (\varvec{u},D,{\hat{\upxi }},{\hat{\varrho }},b)$, this action principle yields the general system

49 $$\begin{aligned} \begin{aligned}&\frac{d}{dt} \frac{\delta \ell }{\delta {\varvec{u}}}+ \pounds _{\varvec{u}}\frac{\delta \ell }{\delta {\varvec{u}}} = D \nabla \frac{\delta \ell }{\delta D} - \Big \langle \frac{\delta \ell }{\delta {\hat{\upxi }}}, \nabla {\hat{\upxi }} \Big \rangle - \frac{\delta \ell }{\delta b} \nabla b - \frac{\delta \ell }{\delta c} \nabla c - \Big \langle \frac{\delta \ell }{\delta {\hat{\varrho }}}, \nabla {\hat{\varrho }} \Big \rangle \\&\frac{d}{dt} \frac{\delta \ell }{\delta {\hat{\upxi }}} + \left[ \frac{\delta \ell }{\delta {\hat{\upxi }}}, {\hat{\upxi }}\right] + {\text {div}} \left( \frac{\delta \ell }{\delta {\hat{\upxi }}}{\varvec{u}}\right) + \left[ {\hat{\varrho }}, \frac{\delta \ell }{\delta {\hat{\varrho }}} \right] =0. \end{aligned} \end{aligned}$$

This system is complemented by the advection equations for *D*, *b*, and *c* given in (32) as well as by the equation for ${{\hat{\varrho }}}$ as in the second of (24). We now consider the Lagrangian (30). In order to simplify the derivation, we note that the last integrand can be written as $\big \langle {{\hat{\varrho }}},i\hbar \varvec{\beta } \cdot \nabla {\hat{\varrho }} \times \nabla {\widehat{H}}\big \rangle$, for the one-form $\varvec{\beta } = c \nabla b$. Hence, the first equation can be equivalently written as

50 $$\begin{aligned} \frac{d}{dt} \frac{\delta \ell }{\delta {\varvec{u}}}+ \pounds _{\varvec{u}}\frac{\delta \ell }{\delta {\varvec{u}}}= & {} D \nabla \frac{\delta \ell }{\delta D} - \left\langle \frac{\delta \ell }{\delta {\hat{\upxi }}}, \nabla {\hat{\upxi }} \right\rangle \nonumber \\{} & {} \quad - \frac{\delta \ell }{\delta \varvec{\beta } } \times {\text {curl}} \varvec{\beta } + \varvec{\beta }{\text {div}} \frac{\delta \ell }{\delta \varvec{\beta } } - \left\langle \frac{\delta \ell }{\delta {\hat{\varrho }}}, \nabla {\hat{\varrho }} \right\rangle \end{aligned}$$

which is obtained with the same variational principle as above, but written in terms of $\varvec{\beta }$, with variations $\delta \varvec{\beta } = {{{\textbf{w}}}\times \nabla \times \varvec{\beta }} - \nabla ( \varvec{\beta } \cdot {{\textbf{w}}})$. The functional derivatives are ${\delta \ell }/{\delta {\varvec{u}}}=MD {\varvec{u}}$, ${\delta \ell }/{\delta {\hat{\upxi }}}=- i \hbar D {\hat{\varrho }}$, and ${\delta \ell }/{\delta \varvec{\beta }}=- \langle {{{\hat{\varrho }}}}, i \hbar \nabla {{\hat{\varrho }}} \times \nabla {{\widehat{H}}} \rangle$, as well as

$$\begin{aligned} \frac{\delta \ell }{\delta D}&= \frac{M}{2} | {\varvec{u}}| ^2 - {\mathcal {E}} (D) - D {\mathcal {E}} '(D) + \langle {{\hat{\varrho }}} | i \hbar {{\hat{\upxi }}} \rangle - \langle {{\hat{\varrho }}} | {{\widehat{H}}} \rangle \\ \frac{\delta \ell }{\delta {\hat{\varrho }}}&= i\hbar D{\hat{\upxi }}-D{{\widehat{H}}} - \frac{1}{2} ic\hbar \{{\hat{\varrho }} , {\widehat{H}} \}_b - \frac{1}{2} ic\hbar \{{\widehat{H}} ,{\hat{\varrho }}\}_b + \partial _k\Big ( \frac{i\hbar }{2} [ \nabla {\widehat{H}}, {{\hat{\varrho }}} ] \times c\nabla b \Big ) _k \,. \end{aligned}$$

Noting the identities

$$\begin{aligned} \partial _k\Big ( {i\hbar } [ \nabla {\widehat{H}}, {{\hat{\varrho }}} ] \times c\nabla b \Big ) _k&=-{i\hbar }c \nabla b \cdot [ \nabla {{\hat{\varrho }}} , \times \nabla {{\widehat{H}}} ] + {i\hbar } [ \{c, {{\widehat{H}}}\}_b, {{\hat{\varrho }}} ] \\&= -{i\hbar } \{ {\hat{\varrho }} , {{\widehat{H}}}\}_b -{i\hbar } \{ {{\widehat{H}}}, {\hat{\varrho }}\}_b+ {i\hbar } [ \{c, {{\widehat{H}}}\}_b, {{\hat{\varrho }}} ], \end{aligned}$$

we can write ${\delta \ell }/{\delta {\hat{\varrho }}}= i\hbar D{\hat{\upxi }}-D{{\widehat{H}}}- i\hbar c \{{\hat{\varrho }}, {\widehat{H}} \}_b - i\hbar c\{{\widehat{H}},{\hat{\varrho }}\}_b+ {i\hbar } [ \{c, {{\widehat{H}}}\}_b, {{\hat{\varrho }}} ]/2$. When inserted in (50) we get

$$\begin{aligned}&D ( \partial _t {\varvec{u}}+ {\varvec{u}}\cdot \nabla {\varvec{u}})\\&\quad = D \nabla \big ( - {\mathcal {E}} (D) - D {\mathcal {E}} '(D) + \langle {{\hat{\varrho }}} \mid i \hbar {{\hat{\upxi }}} \rangle - \langle {{\hat{\varrho }}} \mid {{\widehat{H}}} \rangle \big )\\&\qquad + \langle i \hbar D {\hat{\varrho }}, \nabla {{\hat{\upxi }}} \rangle + \langle {{\hat{\varrho }}} , i \hbar \nabla {\hat{\varrho }} \times \nabla {{\widehat{H}}} \rangle \times {\text {curl}} \varvec{\beta } - \varvec{\beta } {\text {div}} \langle {{\hat{\varrho }}} , i \hbar \nabla {\hat{\varrho }} \times \nabla {{\widehat{H}}} \rangle \\&\qquad - \langle i\hbar D{\hat{\upxi }}-D{{\widehat{H}}}- i\hbar c\{{\hat{\varrho }} , {\widehat{H}} \}_b - i\hbar c\{{\widehat{H}} ,{\hat{\varrho }}\}_b+ \frac{i\hbar }{2} [ \{c, {{\widehat{H}}}\}_b, {{\hat{\varrho }}} ] , \nabla {{\hat{\varrho }}} \rangle \\&\quad =- \nabla {\textsf{p}} - \langle {{\hat{\varrho }}} | D\nabla {{\widehat{H}}} \rangle + \langle {{\hat{\varrho }}} , i \hbar \nabla {\hat{\varrho }} \times \nabla {{\widehat{H}}} \rangle \times {\text {curl}} \varvec{\beta } - \varvec{\beta } {\text {div}} \langle {{\hat{\varrho }}} , i \hbar \nabla {\hat{\varrho }} \times \nabla {{\widehat{H}}} \rangle \\&\qquad + \langle 2 i\hbar c \{{\hat{\varrho }} , {\widehat{H}} \}_b - \frac{i\hbar }{2} [ \{c, {{\widehat{H}}}\}_b, {{\hat{\varrho }}} ] , \nabla {{\hat{\varrho }}} \rangle . \end{aligned}$$

The first equation in (31) is then obtained by noting the following two equalities

$$\begin{aligned}{} & {} \langle {{\hat{\varrho }}},i \hbar \nabla {\hat{\varrho }}\times \nabla \widehat{H}\rangle \times (\nabla c\times \nabla b)- c\nabla b{\text {div}}\langle {{\hat{\varrho }}},i \hbar \nabla {\hat{\varrho }}\times \nabla \widehat{H}\rangle \\{} & {} \quad = \hbar \langle {{\hat{\varrho }}},i \{{{\hat{\varrho }}},{\widehat{H}}\}_b\rangle \nabla c- \hbar {\text {div}}\langle c{{\hat{\varrho }}},i \nabla {\hat{\varrho }}\times \nabla \widehat{H}\rangle \nabla b\\{} & {} \langle 2 i\hbar c \{{\hat{\varrho }}, {\widehat{H}} \}_b - \frac{i\hbar }{2} [ \{c, {{\widehat{H}}}\}_b, {{\hat{\varrho }}} ], \nabla {{\hat{\varrho }}} \rangle = \hbar \langle \nabla {{\hat{\varrho }}},ic\{{{\hat{\varrho }}},{\widehat{H}}\}_b +{i} \{c{{\hat{\varrho }}}, {\widehat{H}}\}_b \rangle . \end{aligned}$$

The second equation in (31) follows directly from the second in (49) by using the expression of the functional derivatives and the advection equation for *D*. Note that the variable ${{\hat{\upxi }}}$ has been eliminated, and that the resulting equation for ${{\hat{\varrho }}}$ is compatible with its advection given in the second of (24).

## Stress Tensor Calculations

We provide here some details concerning the form of the right-hand side of the momentum equation in (35), which involves the sum of two terms: the von Neumann operator term $\langle \widehat{{\mathscr {D}}}, \textrm{d} {\widehat{H}} \rangle$ and the stress tensor term ${\text {div}}{\textsf{T}}$. We also explain the symmetry of ${\textsf{T}}$ as emerging from the rotational invariance of the Lagrangian in terms of $\nabla {\hat{\varrho }}$, $\varvec{\beta } = c \nabla b$ and $\nabla {\widehat{H}}$.

**Structure of the momentum equation.** The situation is best explained by considering the following general form of Lagrangian

51 $$\begin{aligned} \ell (\varvec{u},D,{\hat{\upxi }},{\hat{\varrho }}, \varvec{\beta } )= \int \left[ \frac{M}{2} D|{\varvec{u}}| ^2 - \epsilon \big ({\hat{\upxi }}, D, {\hat{\varrho }}, \nabla {\hat{\varrho }}, \varvec{\beta },{{\widehat{H}}}, \nabla {\widehat{H}}\big ) \right] \textrm{d} ^3q, \end{aligned}$$

which contains (30) as a particular case, with $\varvec{\beta } = c \nabla b$ as before. For this class of Lagrangians, (50) becomes

52 $$\begin{aligned}&{MD} ( \partial _t {\varvec{u}}+ {\varvec{u}}\cdot \nabla {\varvec{u}})\nonumber \\&\quad = {\text {div}} \Big ( \underbrace{- (D \frac{\partial \epsilon }{\partial D} - \epsilon ) \varvec{1} - \frac{\partial \epsilon }{\partial \varvec{\beta } } \otimes \varvec{\beta } - \Big \langle \frac{\partial \epsilon }{\partial \nabla {\hat{\varrho }}} ,\otimes \nabla {\hat{\varrho }} \Big \rangle - \Big \langle \frac{\partial \epsilon }{\partial \nabla {\widehat{H}}}, \otimes \nabla {\widehat{H}}\Big \rangle }_{=-{\textsf{T}} }\Big )\nonumber \\&\qquad + \Big \langle \underbrace{\partial _i \frac{\partial \epsilon }{\partial \partial _i {\widehat{H}}} -\frac{\partial \epsilon }{\partial {\widehat{H}}}}_{=-\widehat{{\mathscr {D}}}= - {\delta \ell }/{\delta {\widehat{H}}} },\nabla {\widehat{H}} \Big \rangle , \end{aligned}$$

which shows the occurrence of a stress tensor ${\textsf{T}}$ and a von Neumann operator, defined in general by $\widehat{{\mathscr {D}}}={\delta \ell }/{\delta {{\widehat{H}}}}$. For our case

$$\begin{aligned} \epsilon ({\hat{\upxi }}, D, {\hat{\varrho }}, \nabla {\hat{\varrho }}, \varvec{\beta }, {{\widehat{H}}}, \nabla {\widehat{H}})= D \big ( {\mathcal {E}} (D)- \big \langle {\hat{\varrho }}\big |i\hbar {\hat{\upxi }}\big \rangle + \big \langle {\hat{\varrho }}\big |{{\widehat{H}}}\big \rangle \big ) +\big \langle {{\hat{\varrho }}},i\hbar \varvec{\beta } \cdot \nabla {\hat{\varrho }} \times \nabla {\widehat{H}}\big \rangle , \end{aligned}$$

one gets

$$\begin{aligned} D\frac{\partial \epsilon }{\partial D} - \epsilon&=D ^2 \frac{\partial {\mathcal {E}} }{\partial D} - \varvec{\beta } \cdot \big \langle {\widehat{{\varvec{\Gamma }}}}, \times \nabla {\widehat{H}}\big \rangle&\left\langle \frac{\partial \epsilon }{\partial \nabla {\hat{\varrho }}}, \otimes \nabla {\hat{\varrho }} \right\rangle&= \left\langle \nabla {\widehat{H}} \times \varvec{\beta } , \otimes \widehat{ \varvec{\Gamma }} \right\rangle \\ \frac{\partial \epsilon }{\partial \varvec{\beta } } \otimes \varvec{\beta }&= \big \langle {\widehat{{\varvec{\Gamma }}}}, \times \nabla {\widehat{H}}\big \rangle \otimes \varvec{\beta }&\left\langle \frac{\partial \epsilon }{\partial \nabla {\widehat{H}}}, \otimes \nabla {\widehat{H}} \right\rangle&= \left\langle \varvec{\beta } \times {\widehat{{\varvec{\Gamma }}}}, \otimes \nabla {\widehat{H}} \right\rangle \end{aligned}$$

so that the stress tensor ${\textsf{T}}$ as defined in (52) gives the expression (36).

**Symmetry of the stress tensor.** We here show how the symmetry of ${\textsf{T}}$ is related to the rotational invariance of the term $\big \langle {{\hat{\varrho }}},i\hbar c\{ {\hat{\varrho }}, {\widehat{H}}\}_b\big \rangle = \big \langle {{\hat{\varrho }}},i\hbar \varvec{\beta } \cdot \nabla {\hat{\varrho }} \times \nabla {\widehat{H}}\big \rangle$ under the transformation $\varvec{\beta } _i \rightarrow \varvec{\beta } _jR^j_i$, $\partial _i {\hat{\varrho }} \rightarrow \partial _j{\hat{\varrho }} R^j_i$, $\partial _i {\widehat{H}} \rightarrow \partial _j{\widehat{H}} R^j_i$, for all $R \in SO(3)$. This again is more easily seen by using the general Lagrangian (51). We consider the following *SO*(3) invariance of $\epsilon$:

53 $$\begin{aligned} \epsilon ({\hat{\upxi }}, D, {\hat{\varrho }}, \partial _j {\hat{\varrho }} R^j_i, \varvec{\beta } _j R^j_i, {{\widehat{H}}}, \partial _j {\widehat{H}}R^j_i)= \epsilon ({\hat{\upxi }}, D, {\hat{\varrho }}, \partial _i {\hat{\varrho }},\varvec{\beta },{{\widehat{H}}}, \partial _i {\widehat{H}}), \end{aligned}$$

for all $R \in SO(3)$. In continuum mechanics, such types of invariance are related to objectivity and material frame indifference (Marsden and Hughes 1983). Taking the derivative with respect to *R* at the identity in the direction $\xi \in \mathfrak {so}(3)$, we have

$$\begin{aligned} \Big ( \frac{\partial \epsilon }{\partial \varvec{\beta } _i} \varvec{\beta } _j + \Big \langle \frac{\partial \epsilon }{\partial \partial _i {\hat{\varrho }} },\partial _j {\hat{\varrho }} \Big \rangle + \Big \langle \frac{\partial \epsilon }{\partial \partial _i {\widehat{H}} }, \partial _j {\widehat{H}} \Big \rangle \Big ) \xi ^j_i=0, \end{aligned}$$

for all $\xi \in \mathfrak {so}(3)$. This is equivalent to the symmetry of $\frac{\partial \epsilon }{\partial \varvec{\beta } } \otimes \varvec{\beta } - \left\langle \frac{\partial \epsilon }{\partial \nabla {\hat{\varrho }}},\otimes \nabla {\hat{\varrho }} \right\rangle - \left\langle \frac{\partial \epsilon }{\partial \nabla {\widehat{H}}}, \otimes \nabla {\widehat{H}}\right\rangle$. Recalling the general definition of ${\textsf{T}}$ in (52), this is equivalent to the symmetry of the tensor ${\textsf{T}}$.

## References

[CR1] Abedi, A., Maitra, N.T., Gross, E.K.U.: Correlated electron-nuclear dynamics: exact factorization of the molecular wavefunction. J. Chem. Phys. **137**(22), 22A530 (2012) 10.1063/1.4745836 23249067

[CR2] Agostini, F., Caprara, S., Ciccotti, G.: Do we have a consistent non-adiabatic quantum-classical mechanics? Eur. Phys. Lett. **78**(3), 30001 (2007)

[CR3] Agostini, F., Min, S.K., Abedi, A., Gross, E.K.U.: Classical-quantum nonadiabatic dynamics: coupled-vs independent-trajectory methods. J. Chem. Theory Comput. **12**(5), 2127–2143 (2016) 27030209 10.1021/acs.jctc.5b01180

[CR4] Akimov, A.V., Long, R., Prezhdo, O.V.: Coherence penalty functional: a simple method for adding decoherence in Ehrenfest dynamics. J. Chem. Phys. **140**, 194107 (2014) 24852530 10.1063/1.4875702

[CR5] Aleksandrov, I.V.: The statistical dynamics of a system consisting of a classical and a quantum subsystem. Z. Naturforsch. **36a**, 902–908 (1981)

[CR6] Bauer, W., Bergold, P., Gay-Balmaz, F., Tronci, C.: Koopmon trajectories in nonadiabatic quantum-classical dynamics. Multiscale Model. Simul. (to appear) (2023). arXiv:2312.13878

[CR7] Baym, G.: Lectures On Quantum Mechanics. CRC Press, Boca Raton (1969)

[CR8] Ben Abdallaha, N., Cáceres, M.J., Carrillo, J.A., Vecil, F.: A deterministic solver for a hybrid quantum-classical transport model in nanoMOSFETs. J. Comput. Phys. **228**, 6553–6571 (2009)

[CR9] Berry, M.V.: Quantal phase factors accompanying adiabatic changes. Proc. R. Soc. A **392**(1802), 45–57 (1984)

[CR10] Bialynicki-Birula, I., Cieplak, M., Karminski, J., Furdyna, A.M.: Theory of Quanta. Oxford University Press, Oxford (1992)

[CR11] Bialynicki-Birula, I., Bialynicka-Birula, Z., Sliwa, C.: Motion of vortex lines in quantum mechanics. Phys. Rev. A **61**(3), 32–110 (2000)

[CR12] Blais, A., Huang, R.-S., Wallraff, A., Girvin, S.M., Schoelkopf, R.J.: Cavity quantum electrodynamics for superconducting electrical circuits: an architecture for quantum computation. Phys. Rev. A **69**(6), 062320 (2004)

[CR13] Bojowald, M., Ding, D.: Canonical description of cosmological backreaction. JCAP **3**, 083 (2021)

[CR14] Bondar, D.I., Gay-Balmaz, F., Tronci, C.: Koopman wavefunctions and classical-quantum correlation dynamics. Proc. R. Soc. A **475**(2229), 20180879 (2019) 31611710 10.1098/rspa.2018.0879 PMC6784396

[CR15] Bondarenko, A.S., Tempelaar, R.: Overcoming positivity violations for density matrices in surface hopping. J. Chem. Phys. **158**(5), 054117 (2023) 36754802 10.1063/5.0135456

[CR16] Boucher, W., Traschen, J.: Semiclassical physics and quantum fluctuations. Phys. Rev. D **37**, 3522–3532 (1988) 10.1103/physrevd.37.3522 9958644

[CR17] Bousquet, D., Hughes, K.H., Micha, D.A., Burghardt, I.: Extended hydrodynamic approach to quantum-classical nonequilibrium evolution. I. Theory. J. Chem. Phys. **134**(6), 064116 (2011) 21322670 10.1063/1.3553174

[CR18] Burghardt, I., Bagchi, B.: On the non-adiabatic dynamics of solvation: a molecular hydrodynamic formulation. Chem. Phys. **329**, 343–356 (2006)

[CR19] Burghardt, I., Parlant, G.: On the dynamics of coupled Bohmian and phase-space variables: a new hybrid quantum-classical approach. J. Chem. Phys. **120**(7), 3055–3058 (2004) 15268457 10.1063/1.1647059

[CR20] Calkin, M.G.: An action principle for magnetohydrodynamics. Can. J. Phys. **41**, 2241 (1963)

[CR21] Chattaraj, P.K. (ed.): Quantum Trajectories. CRC Press, Boca Raton (2011)

[CR22] Chruściński, D., Kossakowski, A., Marmo, G., Sudarshan, E.C.G.: Dynamics of interacting classical and quantum systems. Open. Syst. Inf. Dyn. **18**(4), 339–351 (2011)

[CR23] Crespo-Otero, R., Barbatti, M.: Recent advances and perspectives on nonadiabatic mixed quantum-classical dynamics. Chem. Rev. **118**(15), 7026–7068 (2018) 29767966 10.1021/acs.chemrev.7b00577

[CR24] Curchod, B.F.E., Tavernelli, I., Rothlisberger, U.: Trajectory-based solution of the nonadiabatic quantum dynamics equations: an on-the-fly approach for molecular dynamics simulations. Phys. Chem. Chem. Phys. **13**(8), 3231–3236 (2011) 21264437 10.1039/c0cp02175j

[CR25] de Gennes, P.G.: Short range order effects in the isotropic phase of nematics and cholesterics. Mol. Cryst. Liq. Cryst. **12**, 193–214 (1971)

[CR26] Degond, P., Gallego, S., Méhats, F.: Isothermal quantum hydrodynamics: derivation, asymptotic analysis, and simulation. Multiscale Model. Simul. **6**(1), 246–272 (2007)

[CR27] Degond, P., Gallego, S., Méhats, F.: On quantum hydrodynamic and quantum energy transport models. Commun. Math. Sci. **5**(4), 887–908 (2007)

[CR28] Diósi, L.: Hybrid quantum-classical master equations. Phys. Scr. **T163**, 014004 (2014)

[CR29] Dirac, P.A.M.: Quantised singularities in the electromagnetic field. Proc. R. Soc. A **133**(821), 60–72 (1931)

[CR30] Fang, D., Jin, S., Sparber, C.: An efficient time-splitting method for the Ehrenfest dynamics. Multiscale Model. Simul. **16**, 900–921 (2018)

[CR31] Feynman, R.: Forces in molecules. Phys. Rev. **56**(4), 340–343 (1939)

[CR32] Foskett, M.S., Tronci, C.: Holonomy and vortex structures in quantum hydrodynamics. In: Fathi, A., Morrison, P.J., M-Seara, T., Tabachnikov, S. (eds.) Hamiltonian Systems: Dynamics, Analysis, Applications. Mathematical Sciences Research Institute, vol. 72. Cambridge University Press, Cambridge (2024)

[CR33] Foskett, M.S., Holm, D.D., Tronci, C.: Geometry of nonadiabatic quantum hydrodynamics. Acta Appl. Math. **162**, 1–41 (2019)

[CR34] Gamba, I., Jüngel, A.: Asymptotic limits for quantum trajectory models. Commun. Partial Differ. Equs. **7**, 669–691 (2002)

[CR35] Gamba, I.M., Jüngel, A., Vasseur, A.: Global existence of solutions to one-dimensional viscous quantum hydrodynamic equations. J. Differ. Equ. **247**, 3117–3135 (2009)

[CR36] Garashchuk, S., Rassolov, V., Prezhdo, O.: Semiclassical Bohmian dynamics. Rev. Comput. Chem. **27**, 287–368 (2010)

[CR37] Garashchuk, S., Stetzler, J., Rassolov, V.: Factorized electron-nuclear dynamics with an effective complex potential. J. Chem. Theory Comput. **19**(5), 1393–1408 (2023) 36795898 10.1021/acs.jctc.2c01019

[CR38] Gawlik, E.S., Gay-Balmaz, F.: A variational finite element discretization of compressible flow. Found. Comput. Math. **21**, 961–1001 (2021)

[CR39] Gay-Balmaz, F., Ratiu, T.S.: The geometric structure of complex fluids. Adv. Appl. Math. **42**(2), 176–275 (2009)

[CR40] Gay-Balmaz, F., Tronci, C.: Madelung transform and probability densities in hybrid quantum-classical dynamics. Nonlinearity **33**(10), 5383–5424 (2019)

[CR41] Gay-Balmaz, F., Tronci, C.: Evolution of hybrid quantum-classical wavefunctions. Phys. D **440**, 133450 (2022)

[CR42] Gay-Balmaz, F., Tronci, C.: Koopman wavefunctions and classical states in hybrid quantum-classical dynamics. J. Geom. Mech. **14**(4), 559–596 (2022)

[CR43] Gerasimenko, V.: Dynamical equations of quantum-classical systems. Theor. Math. Phys. **50**, 49–55 (1982)

[CR44] Gu, B., Franco, I.: Partial hydrodynamic representation of quantum molecular dynamics. J. Geom. Chem. **146**, 194104 (2017) 10.1063/1.4983495 PMC5648576 28527433

[CR45] Hall, M.J.W., Reginatto, M.: Ensembles on Configuration Space. Springer, Berlin (2016)

[CR46] Henyey, F.: Gauge groups and Noether’s theorem for continuum mechanics. AlP Conf. Proc. **88**, 85–90 (1982)

[CR47] Holm, D.D.: Euler–Poincaré dynamics of perfect complex fluids. In: Holmes, P., Newton, P., Weinstein, A. (eds.) Geometry, Mechanics, and Dynamics. Springer, New York (2002)

[CR48] Holm, D.D., Kupershmidt, B.A.: Hamiltonian formulation of ferromagnetic hydrodynamics. Phys. Lett. A **129**, 93–100 (1988)

[CR49] Holm, D.D., Marsden, J.E., Ratiu, T.S., Weinstein, A.: Nonlinear stability of fluid and plasma equilibria. Phys. Rep. **123**, 1–116 (1985)

[CR50] Holm, D.D., Marsden, J.E., Ratiu, T.S.: The Euler–Poincaré equations and semidirect products with applications to continuum theories. Adv. Math. **137**, 1–81 (1998)

[CR51] Holm, D.D., Rawlinson, J.I., Tronci, C.: The bohmion method in nonadiabatic quantum hydrodynamics. J. Phys. A Math. Theor. **54**, 495201 (2021)

[CR52] Hughes, K.H., Parry, S.M., Burghardt, I.: Closure of quantum hydrodynamic moment equations. J. Chem. Phys. **130**(5), 054115 (2009) 19206966 10.1063/1.3073759

[CR53] Hughes, K.H., Baxter, S.N., Bousquet, D., Ramanathan, P., Burghardt, I.: Extended hydrodynamic approach to quantum-classical nonequilibrium evolution. II. Application to nonpolar solvation. J. Chem. Phys. **136**(1), 014102 (2012) 22239764 10.1063/1.3671378

[CR54] Hurst, J., Hervieux, P.-A., Manfredi, G.: Phase-space methods for the spin dynamics in condensed matter systems. Phil. Trans. R. Soc. A **375**, 20160199 (2017) 28320903 10.1098/rsta.2016.0199 PMC5360899

[CR55] Kapral, R.: Progress in the theory of mixed quantum-classical dynamics. Annu. Rev. Phys. Chem. **57**, 129–57 (2006) 16599807 10.1146/annurev.physchem.57.032905.104702

[CR56] Kendrick, B.K.: A new method for solving the quantum hydrodynamic equations of motion. J. Chem. Phys. **119**(12), 5805–5817 (2003) 10.1063/1.1630302 15267894

[CR57] Koopman, B.O.: Hamiltonian systems and transformations in Hilbert space. Proc. Nat. Acad. Sci. **17**, 315 (1931) 16577368 10.1073/pnas.17.5.315 PMC1076052

[CR58] Madelung, E.: Quantentheorie in hydrodynamischer Form. Z. Phys. **40**(3–4), 322–326 (1927)

[CR59] Manfredi, G., Rittaud, A., Tronci, C.: Hybrid quantum-classical dynamics of pure-dephasing systems. J. Phys. A Math. Theor. **56**(15), 154002 (2023)

[CR60] Marsden, J.E., Hughes, T.J.R.: Mathematical Foundations of Elasticity. Prentice Hall, New York (1983). (reprinted by Dover, New York, 1994)

[CR61] Mead, A.C.: The geometric phase in molecular systems. Rev. Mod. Phys. **64**(1), 51–85 (1992)

[CR62] Nambu, Y.: Generalized Hamiltonian dynamics. Phys. Rev. D **7**(8), 2405–2412 (1973)

[CR63] Petrović, M.D., Popescu, B.S., Bajpai, U., Plecháč, P., Nikolić, B.K.: Spin and charge pumping by a steady or pulse-current-driven magnetic domain wall: a self-consistent multiscale time-dependent quantum-classical hybrid approach. Phys. Rev. App. **10**(5), 054038 (2018)

[CR64] Reichman, D., Silbey, R.J., Suárez, A.: On the nonperturbative theory of pure dephasing in condensed phases at low temperatures. J. Chem. Phys. **105**(23), 10500–10506 (1996)

[CR65] Rosspeintner, A., Lang, B., Vauthey, E.: Ultrafast photochemistry in liquids. Ann. Rev. Phys. Chem. **64**, 247–271 (2013) 23298248 10.1146/annurev-physchem-040412-110146

[CR66] Santoro, F., Green, J.A., Martinez-Fernandez, L., Cerezo, J., Improta, R.: Quantum and semiclassical dynamical studies of nonadiabatic processes in solution: achievements and perspectives. Phys. Chem. Chem. Phys. **23**(14), 8181 (2021) 33875988 10.1039/d0cp05907b

[CR67] Sudarshan, E.C.G.: Interaction between classical and quantum systems and the measurement of quantum observables. Prāmaṇa **6**(3), 117–126 (1976)

[CR68] Suzuki, Y., Watanabe, K.: Bohmian mechanics in the exact factorization of electron-nuclear wave functions. Phys. Rev. A **94**(3), 032517 (2016)

[CR69] Thomas, L.H.: The kinematics of an electron with an axis. Phil. Mag. **7**, 1–23 (1927)

[CR70] Tronci, C.: Hybrid models for perfect complex fluids with multipolar interactions. J. Geom. Mech. **4**(3), 333–363 (2012)

[CR71] Tronci, C., Gay-Balmaz, F.: Lagrangian trajectories and closure models in mixed quantum-classical dynamics. Lecture Notes Comput. Sci. **14072**, 290–300 (2023)

[CR72] Tronci, C., Tassi, E., Morrison, P.J.: Energy-Casimir stability of hybrid Vlasov-MHD models. J. Phys. A Math. Theor. **48**, 185501 (2015)

[CR73] Tully, J.C.: Nonadiabatic dynamics. In: Thompson, D.L. (ed.) Modern Methods for Multidimensional Dynamics Computations in Chemistry. World Scientific, Singapore (1998)

[CR74] Vaisman, I.: A survey on Nambu–Poisson brackets. Acta Math. Univ. Comen. **68**(2), 213–241 (1999)

[CR75] van Hove, L.: On Certain Unitary Representations of an Infinite Group of Transformations. PhD Thesis. Word Scientific 2001 (1951)

[CR76] Wu, Y., Bian, X., Rawlinson, J.I., Littlejohn, R.G., Subotnik, J.E.: A phase-space semiclassical approach for modeling nonadiabatic nuclear dynamics with electronic spin. J. Chem. Phys. **157**(1), 011101 (2022) 35803809 10.1063/5.0093345

[CR77] Wyatt, R.E.: Quantum Dynamics with Trajectories: Introduction to Quantum Hydrodynamics. Springer, Berlin (2005)

[CR78] Zhao, Y., Makri, N.: Bohmian versus semiclassical description of interference phenomena. J. Chem. Phys. **119**(1), 60–67 (2003)

[CR79] Zimmermann, T., Vaníček, J.: Measuring nonadiabaticity of molecular quantum dynamics with quantum fidelity and with its efficient semiclassical approximation. J. Chem. Phys. **136**(9), 094106 (2012) 22401428 10.1063/1.3690458

